# 14-3-3**ζ** Constrains insulin secretion by regulating mitochondrial function in pancreatic **β** cells

**DOI:** 10.1172/jci.insight.156378

**Published:** 2022-04-22

**Authors:** Yves Mugabo, Cheng Zhao, Ju Jing Tan, Anindya Ghosh, Scott A. Campbell, Evgenia Fadzeyeva, Frédéric Paré, Siew Siew Pan, Maria Galipeau, Julia Ast, Johannes Broichhagen, David J. Hodson, Erin E. Mulvihill, Sophie Petropoulos, Gareth E. Lim

**Affiliations:** 1 Department of Medicine, Université de Montréal, Montreal, Quebec, Canada.; 2 Cardiometabolic axis, Centre de Recherche du Centre hospitalier de l’Université de Montréal (CRCHUM), Montreal, Quebec, Canada.; 3 Division of Obstetrics and Gynecology, Department of Clinical Science, Intervention and Technology, Karolinska Institutet and Karolinska University Hospital, Stockholm, Sweden.; 4 Immunopathology axis, CRCHUM, Montreal, Quebec, Canada.; 5 The University of Ottawa, Faculty of Medicine, Department of Biochemistry, Microbiology and Immunology, Ottawa, Ontario, Canada.; 6 The University of Ottawa Heart Institute, Ottawa, Ontario, Canada.; 7 Institute of Metabolism and Systems Research (IMSR), Centre of Membrane Proteins and Receptors (COMPARE), University of Birmingham, Birmingham, United Kingdom.; 8 Centre for Endocrinology, Diabetes and Metabolism, Birmingham Health Partners, Birmingham, United Kingdom.; 9 Leibniz-Forschungsinstitut für Molekulare Pharmakologie (FMP), Department of Chemical Biology, Berlin, Germany.

**Keywords:** Endocrinology, Metabolism, Adaptor proteins, Beta cells, Mitochondria

## Abstract

While critical for neurotransmitter synthesis, 14-3-3 proteins are often assumed to have redundant functions due to their ubiquitous expression, but despite this assumption, various 14-3-3 isoforms have been implicated in regulating metabolism. We previously reported contributions of 14-3-3ζ in β cell function, but these studies were performed in tumor-derived MIN6 cells and systemic KO mice. To further characterize the regulatory roles of 14-3-3ζ in β cell function, we generated β cell–specific 14-3-3ζ–KO mice. Although no effects on β cell mass were detected, potentiated glucose-stimulated insulin secretion (GSIS), mitochondrial function, and ATP synthesis were observed. Deletion of 14-3-3ζ also altered the β cell transcriptome, as genes associated with mitochondrial respiration and oxidative phosphorylation were upregulated. Acute 14-3-3 protein inhibition in mouse and human islets recapitulated the enhancements in GSIS and mitochondrial function, suggesting that 14-3-3ζ is the critical isoform in β cells. In dysfunctional *db/db* islets and human islets from type 2 diabetic donors, expression of *Ywhaz*/*YWHAZ*, the gene encoding 14-3-3ζ, was inversely associated with insulin secretion, and pan–14-3-3 protein inhibition led to enhanced GSIS and mitochondrial function. Taken together, this study demonstrates important regulatory functions of 14-3-3ζ in the regulation of β cell function and provides a deeper understanding of how insulin secretion is controlled in β cells.

## Introduction

The downstream signaling events underlying glucose-stimulated insulin secretion (GSIS) are well conserved in rodent and human β cells. Following the uptake of glucose into pancreatic β cells by low-affinity transporters, it is converted into pyruvate before entering the tricarboxylic acid (TCA) cycle and oxidative phosphorylation cascade to yield ATP (1). Increases in the ATP/ADP ratio then lead to closure of K-ATP channels to depolarize the β cell and the subsequent opening of voltage-dependent Ca^2+^ channels. Along with amplifying signals, the rise in intracellular Ca^2+^ drives insulin granule exocytosis. Insulin secretion follows 2 phases: a rapid, maximal release period of insulin release, followed by a lower-magnitude, sustained second phase of insulin secretion (2). Aside from causing the closure of K-ATP channels, ATP is also required for kinesin-mediated translocation of latent insulin granules to the plasma membrane for the second phase of insulin secretion (3). These events require precise spatial and temporal coordination to ensure proper insulin release to maintain normoglycemia (4).

Scaffold proteins are essential regulators of signaling events due to their ability to facilitate the translocation of downstream effectors and influence the activities of receptors, kinases, and enzymes (5, 6). In β cells, various scaffolds, such as β-arrestin-2, AKAP150, and NCK1, have been found to be necessary for insulin secretion (7–9). For example, β cell–specific deletion or siRNA-mediated knockdown of β-arrestin-2 in mouse β cells or EndoC-βH1 cells, respectively, results in defective GSIS (9). Additionally, islets from systemic AKAP150-KO mice display impaired insulin secretion due to alterations in Ca^2+^-evoked currents (7).

The 14-3-3 proteins are a ubiquitously expressed family of scaffolds that were initially discovered in brain extracts, and they compose approximately 1% of soluble proteins in the brain (5, 6). Their ability to recognize serine phosphorylated or threonine phosphorylated proteins results in a large interactome that is shared among all 7 mammalian isoforms (10–12). Despite exhibiting high-sequence homology, isoform-specific roles of 14-3-3 proteins have been identified. We previously identified 14-3-3ζ as a critical regulator of adipogenesis, glucose homeostasis, and pancreatic β cell survival (13–16). Systemic deletion of 14-3-3ζ in mice was associated with significant reductions in adipogenesis, and siRNA-mediated depletion of only 14-3-3ζ in 3T3-L1 preadipocytes abrogated adipocyte differentiation (13). With respect to glucose homeostasis, whole-body 14-3-3–KO mice displayed enhanced oral glucose tolerance due to significantly elevated fasting levels of the incretin hormone, GLP-1 (15).

One of the earliest indications of 14-3-3 proteins being involved in hormone secretion was identified by Morgan and Burgoyne, who found that 14-3-3 proteins participate in calcium-dependent release of catecholamines from bovine adrenal chromaffin cells (17). The contribution of 14-3-3 proteins in exocytosis is also conserved across organisms, as the *Drosophila* homolog of 14-3-3ζ, Leonardo, was discovered to regulate the dynamics of synaptic vesicles and synaptic transmission rates (18). We first explored the possibility of 14-3-3 proteins to influence insulin secretion, and plasmid-based overexpression of difopein, a 14-3-3 protein inhibitor, in dispersed mouse islets or MIN6 cells attenuated insulin secretion. However, this was also associated with increased cell death, an event known to be associated with impaired insulin secretion (14, 19–21). We followed this by depleting individual isoforms by siRNA in MIN6 cells and found no impact on insulin secretion, whereas overexpression of HA-14-3-3ζ attenuated GSIS (15). Depletion of 14-3-3ζ by siRNA or overexpression was found to complimentarily induce MIN6 cell apoptosis or promote survival, respectively (14). Interestingly systemic 14-3-3ζ–KO mice had increased β cell area, which suggested proliferative actions of 14-3-3ζ in the β cell (15). With these conflicting results from MIN6 insulinoma cells and whole-body 14-3-3ζ–KO mice, it is unclear whether 14-3-3ζ has cell-autonomous roles in β cells to influence insulin secretion, cell survival, or proliferation. Thus, in-depth studies are required to truly understand the β cell–specific roles of 14-3-3ζ.

Using a combination of approaches and models, we identify 14-3-3ζ as a critical regulator of insulin secretion in primary β cells. Deletion of 14-3-3ζ specifically in β cells enhanced GSIS, demonstrating a physiological role of 14-3-3ζ to constrain insulin secretion. Single-cell RNA-Seq revealed significant upregulation of genes and pathways associated with mitochondrial respiration and oxidative phosphorylation in 14-3-3–deficient β cells, which were reflected by changes in mitochondrial mass and activity. Acute inhibition of all 14-3-3 proteins in primary mouse and human islets recapitulated the potentiation of GSIS and mitochondrial function that occurred following 14-3-3ζ deletion in β cells, suggesting that 14-3-3ζ could be a key isoform in human β cells. Expression levels of *Ywhaz/YWHAZ*, the gene encoding 14-3-3ζ, were found to be inversely associated with insulin secretory capacity and to be significantly elevated in dysfunctional *db/db* islets and human islets from type 2 diabetic donors, and inhibition of 14-3-3 proteins was able to alleviate β cell dysfunction. Taken together, this study unequivocally demonstrates the ability of 14-3-3ζ to restrain insulin release from pancreatic β cells by influencing mitochondrial mass and function. Moreover, it further deepens our knowledge of the regulatory factors present in a β cell that control insulin secretion.

## Results

### Inhibition of 14-3-3 proteins is sufficient to increase insulin secretion.

**Figure 1:**
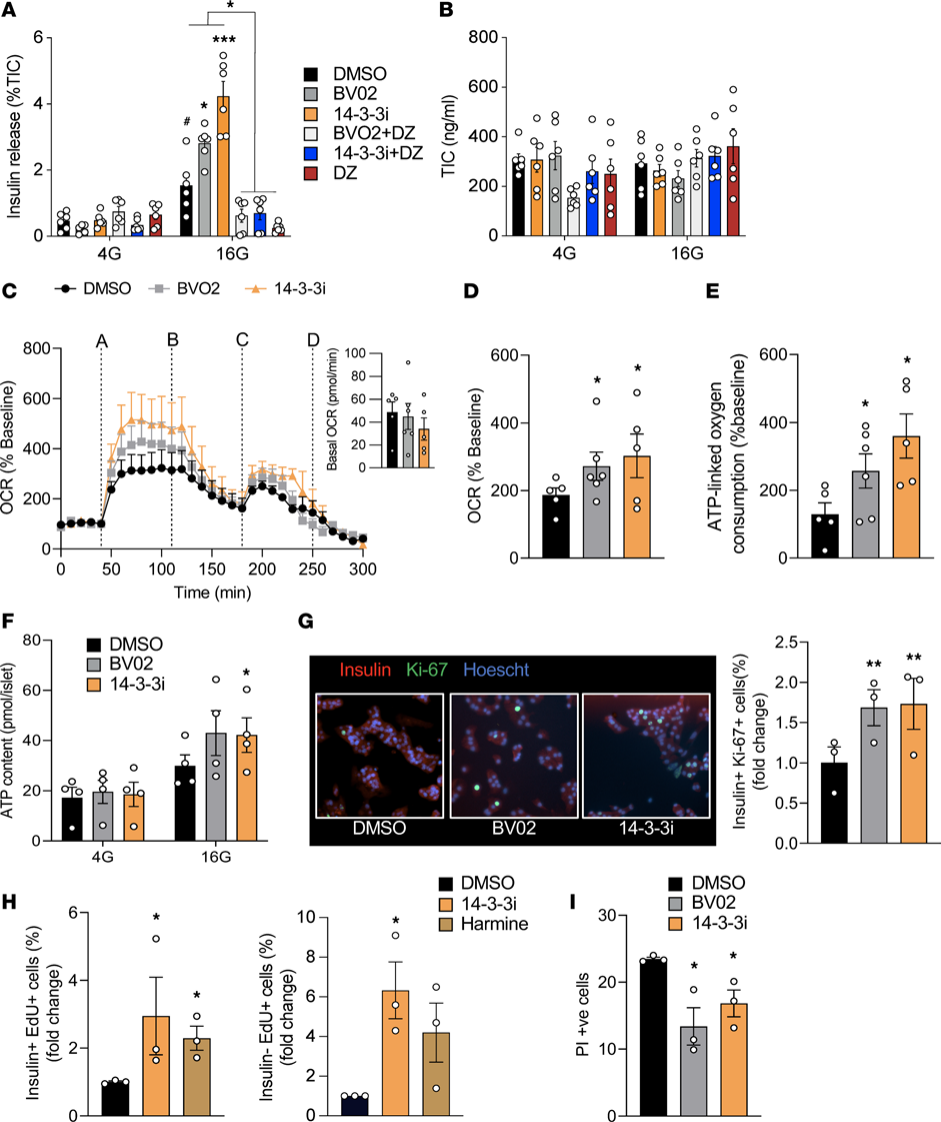
14-3-3 Protein inhibition in mouse islets enhances insulin secretion, mitochondrial function, and proliferation. (**A** and **B**) Mouse islets were incubated with 14-3-3 inhibitors (10 μM) and diazoxide (DZ, 200 μM) for 1 hour prior to 4 (4G) or 16 (16G) mM glucose for 1 hour. Insulin secretion was measured by radioimmunoassay (**A**) and normalized to total insulin content (**B**; *n* = mice 5–6 per group; ^#^*P* < 0.05 versus DMSO 4G; *P < 0.05 and ***P < 0.001 versus DMSO 16G). (**C**) Combined OCR trace, with basal OCRs in the inset image, showing when islets were treated with (line A) 16 mM glucose, (line B) oligomycin (5 μM), (line C) FCCP (1 μM), and (line D) rotenone (5 μM) and antimycin (5 μM). (**D** and **E**) Glucose-induced OCR (**D**) and ATP-linked oxygen consumption (**E**) were measured (*n* = 5–6 mice per group; *P < 0.05 versus DMSO). (**F**) Biochemical ATP measurements in islets treated with 14-3-3 inhibitors (*n* = 4 mice per group; *P < 0.05). (**G**) In dispersed islets, β cell proliferation was measured by immunostaining for insulin^+^ and Ki-67^+^ β cells after 72-hour treatment with 14-3-3 inhibitors (*n* = 3 per group; *P < 0.05). (**H**) β Cell proliferation was quantified by flow cytometry–mediated detection of insulin^+^ and EdU^+^ β cells, following 72-hour treatment with 14-3-3i or harmine (10 μM each). Insulin^–^ and EdU^+^ cells were also measured (*n* = 3 per group; *P < 0.05). (**I**) Cell death, defined by propidium iodide^+^ (PI, 0.5 μg/mL) and Hoechst 33342^+^ (50 ng/mL) cells, was measured in dispersed islets exposed to 14-3-3 inhibitors (10 μM each) for 72 hours (*n* = 3 per group; *P < 0.05). Significance was determined by 1-way ANOVA, followed by Dunnett’s test (**C**–**E** and **G**–**I**), or 2-way ANOVA, followed by Tukey’s test (**A** and **F**).

**Figure 2:**
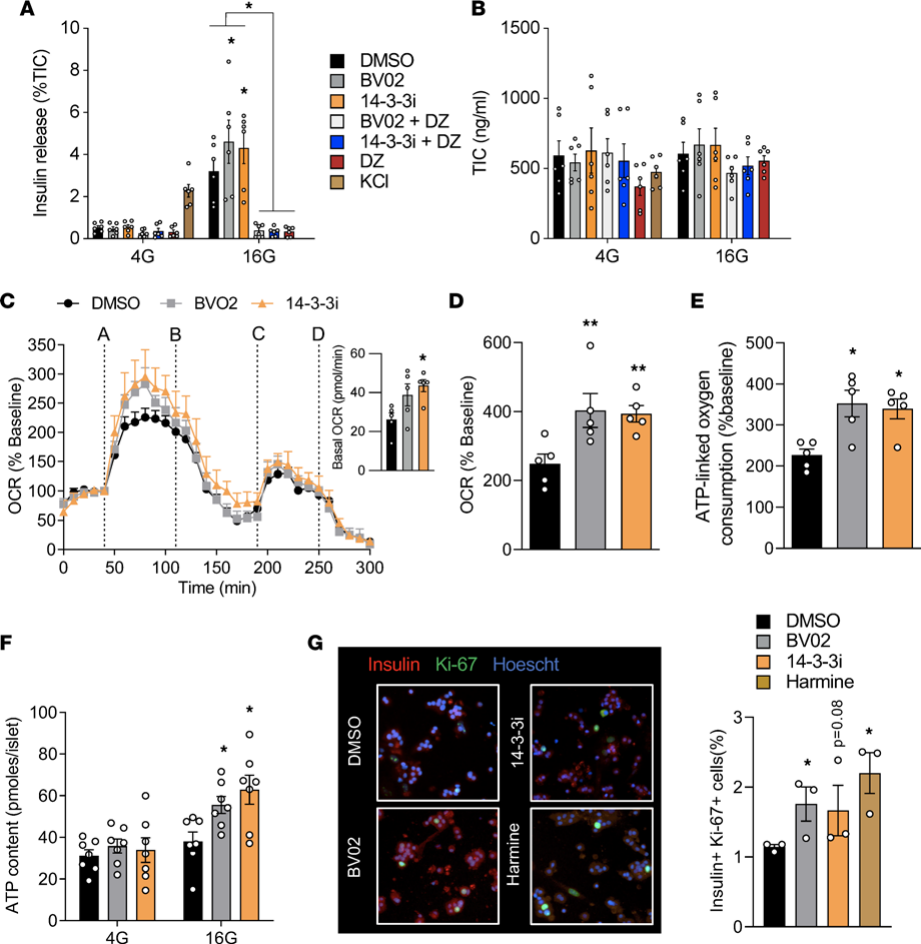
Pharmacological inhibition of 14-3-3 proteins in human pancreatic islets enhances insulin secretion, mitochondrial function, and proliferation. (**A**) Human islets were incubated with 2 pan–14-3-3 inhibitors (14-3-3i and BVO2; 10 μM each) plus or minus diazoxide (DZ, 200μM) for 1 hour at 4 mM glucose prior to treatment with 4 (4G) or 16 (16G) mM glucose for 1 hour. Insulin secretion was measured by radioimmunoassay and normalized to total insulin content (*n* = 5–6 donors per group; ^#^*P* < 0.05 when compared with DMSO 4 G; *P < 0.05, ***P < 0.001 when compared with DMSO 16G). (**B**) Quantification of total insulin content (TIC) in acid-ethanol extracts from each (n = 5–6 donors per group). (**C**) Combined OCR trace, with basal OCRs in the inset image, showing when islets were treated with (line A) 16 mM glucose, (line B) oligomycin (5 μM), (line C) FCCP (1 μM), and (line D) rotenone (5 μM) and antimycin (5 μM). (**D** and **E**) OCR in response to glucose (**D**) and ATP-linked oxygen consumption (**E**) were measured. ATP-linked OCR was calculated by measuring the decrease in OCR upon injection of oligomycin. (*n* = 5–6 donors per group; *P < 0.05 when compared with DMSO). (**F**) Biochemical measurements of ATP content in isolated mouse islets treated with 14-3-3 inhibitors and quantified at different glucose concentrations (*n* = 7 donors per group; *P < 0.05). (**G**) In dispersed mouse islet preparations, β cell proliferation, as measured by immunofluorescent staining for Insulin^+^ and Ki-67^+^ β cells, was measured after 72-hour treatment with DMSO, 14-3-3 inhibitors (10 μM each), or harmine (10 μM) (*n* = 3 donors per group; *P < 0.05). Significance was determined by 1-way ANOVA, followed by Dunnett’s test (**C**–**E** and **G**), or by 2-way ANOVA, followed by Tukey’s multiple-comparison test (**A** and **F**).

We previously reported that overexpression of the 14-3-3 protein peptide inhibitor, difopein, in MIN6 cells and dispersed mouse islets impaired GSIS, in addition to inducing cell death (14, 22). Given the importance of cell-to-cell contact in propagating signals underlying insulin secretion (23, 24) and given the differences in insulin secretion between cell lines and primary β cells (25), intact mouse and human islets were treated with cell-permeable 14-3-3 inhibitors, 14-3-3i and BV02 (26–28). Pan-inhibition was found to potentiate GSIS (Figure 1A and Figure 2A). Opening of K-ATP channels with diazoxide was sufficient to prevent the potentiation of GSIS due to 14-3-3 inhibitors, demonstrating that the effects of 14-3-3 protein inhibition on GSIS were upstream to that of K-ATP channel closure in mouse and human islets (Figure 1A and Figure 2A). To address whether inhibition of 14-3-3 proteins altered the responsiveness of β cells to glucose, normal mouse islets were exposed to different concentrations of glucose in the absence or presence of 14-3-3i or BV02. Inhibition of 14-3-3 proteins lowered the glucose threshold to stimulate GSIS such that 10 mM of glucose was sufficient to stimulate insulin secretion (Supplemental Figure 1A; supplemental material available online with this article; https://doi.org/10.1172/jci.insight.156378DS1). Potentiated GSIS was observed following 14-3-3 protein inhibition at each test concentration of glucose, but no concentration of glucose was able to exceed the maximal amount of secreted insulin in response to 25 mM glucose (Supplemental Figure 1A). Acute inhibition of 14-3-3 proteins by 14-3-3i or BV02 did not affect total insulin content (TIC) (Figure 1B and Figure 2B).

One of the key events underlying GSIS is the generation of ATP in mitochondria, and it is needed to propagate downstream signals involved in insulin release (1). In plant cells and mouse platelets, 14-3-3 proteins have been shown to inhibit mitoplast ATP synthase activity and mitochondrial reserve capacity, respectively (29, 30), but whether 14-3-3ζ has similar roles in mouse or human β cells is not known. Inhibition of 14-3-3 proteins in intact mouse and human islets increased glucose-induced mitochondrial activity, as measured by oxygen consumption rates (OCR) (Figure 1, C and D, and Figure 2, C and D). Moreover, the increase in OCR was associated with increased ATP-linked oxygen consumption, which can be a surrogate measure of ATP synthesis (Figure 1E and Figure 2E). To further confirm changes in ATP synthesis, total ATP levels in mouse and human islets exposed to low and high glucose were measured, and significantly enhanced ATP synthesis was detected in the presence of 14-3-3 protein inhibitors (Figure 1F and Figure 2F). When taken together, these findings demonstrate that 14-3-3 proteins have inhibitory effects on GSIS due, in part, to restriction of mitochondrial function and ATP synthesis.

### Pan-inhibition of 14-3-3 proteins increases β cell proliferation.

Cell type–dependent contributions of 14-3-3 proteins on proliferation have been reported. For example, in U2OS cells, genetic inhibition of 14-3-3 proteins have been shown to allow cells to prematurely enter the cell cycle and proliferate (31). In contrast, overexpression of some 14-3-3 isoforms in cancer cells are linked to proliferation and increased cell survival (14, 32–34). To determine if 14-3-3 proteins regulates β cell proliferation, 2 independent measurements of proliferation were used. Firstly, dispersed mouse and human islets were exposed to 14-3-3i or BV02 for 72 hours, which led to significant increases in β cell proliferation, as measured by the percentage of Ki-67^+^ and insulin^+^ cells (Figure 1G and Figure 2G). Secondly, mouse islets were incubated with 14-3-3i and harmine for 72 hours in the presence of EdU, and flow cytometry confirmed the ability of 14-3-3i to induce β cell proliferation, in addition to insulin^–^ cells (Figure 1H). Harmine has been shown to promote β cell proliferation by inhibiting DYRK1A (35). Cell type–dependent effects on cell survival have been observed following the overexpression or depletion of various 14-3-3 proteins (22, 36, 37), and while we previously reported that 14-3-3 protein inhibition or siRNA-mediated knockdown of 14-3-3 isoforms induced apoptosis in MIN6 insulinoma cells (14), incubation of mouse islet cells with 14-3-3i or BV02 for 72 hours did not have detrimental effects on cell viability (Figure 1I).

### Deletion of 14-3-3ζ in murine β cells enhances insulin secretion.

We previously characterized the metabolic phenotype of systemic 14-3-3ζ–KO mice and found that whole-body deletion of 14-3-3ζ was associated with improved oral glucose tolerance due to increased circulating levels of the incretin hormone GLP-1 (15). Moreover, loss of 14-3-3ζ was associated with significantly increased β cell area, potentially due to compensatory β cell expansion to account for decreased insulin sensitivity (15).

**Figure 3:**
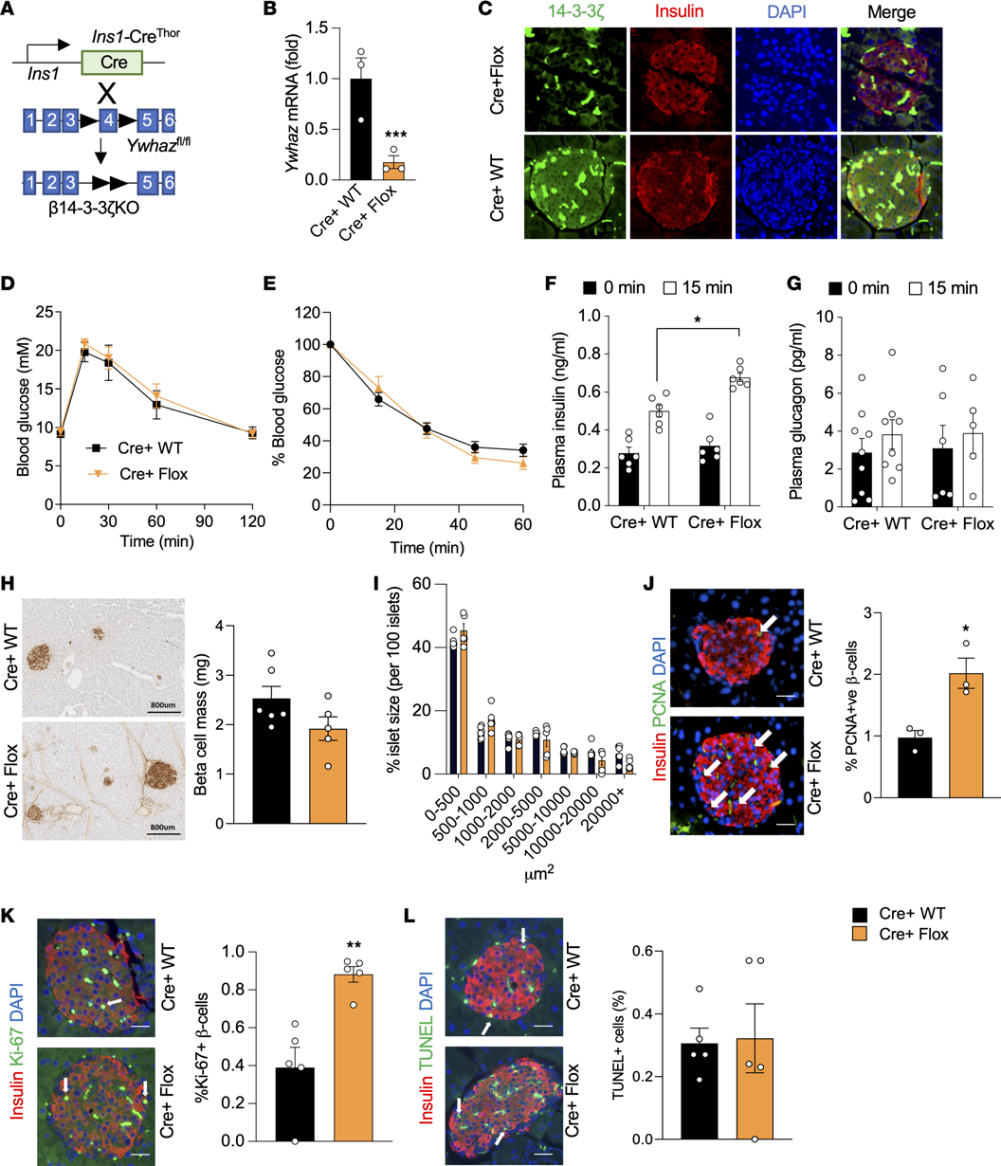
β Cell–specific deletion of 14-3-3ζ enhances glucose-induced insulin secretion in vivo and increases β cell proliferation. (**A**) Generation of β cell–specific 14-3-3–KO mice (Cre^+^ Flox) was accomplished by breeding *Ins1*Cre^Thor^ mice with mice harboring floxed alleles of *Ywhaz*. (**B**) Isolated mRNA from islets from Cre^+^ Flox mice and their littermate controls (Cre^+^ WT) were subjected to qPCR analysis for *Ywhaz* mRNA levels (*n =* 3 per genotype; ***P < 0.001) (**C**) Immunofluorescence staining for insulin and 14-3-3ζ on Cre^+^ WT and Cre^+^ Flox pancreatic sections (representative images of *n =* 3 mice per genotype). Magnification ×20. Scale bar = 100 μm. (**D** and **E**) No differences in glucose (**D**) or insulin (**E**) tolerance were observed in Cre^+^ Flox mice following i.p. injections of glucose (2 g/kg) or insulin (0.75 IU/kg), respectively (*n =* 5–9 mice per genotype). (**F** and **G**) Cre^+^ Flox mice displayed potentiated insulin secretion (**F**) following i.p. glucose (2 g/kg) injections, and no differences were observed in circulating glucagon (**G**) (*n =* 5–9 per genotype; *P < 0.05). (**H** and **I**) Pancreatic tissue from 12-week-old Cre^+^ WT and Cre^+^ Flox mice were collected, and β cell mass (**H**) and islet size distribution (**I**) were determined (*n =* 5–6 mice, 4 sections per mouse). Scale bar: 800 μm. (**J** and **K**) β Cell proliferation was measured by coimmunostaining for PCNA^+^ (**J**, *n =* 3 per genotype; *P < 0.05; scale bar: 100 μm) or Ki-67^+^ β cells (**K**, *n =* 3 per genotype; **P < 0.01; scale bar: 100 μm). White arrows denote positive cells. (**L**) TUNEL^+^ apoptotic β cells (white arrows) were measured in 4 pancreatic sections from Cre^+^ WT and Cre^+^ Flox mice. Scale bar: 100 μm. White arrows denote positive cells. Significance was determined by unpaired, 2-tailed Student’s *t* test (**B**, **J**, **K**, and **L**) or by 2-way ANOVA, followed by Tukey’s multiple-comparison test (**F**).

To better understand how 14-3-3ζ influences β cell function, β cell–specific 14-3-3ζ–KO mice (Cre^+^ Flox or β14-3-3ζ–KO) were generated by breeding 14-3-3ζ–floxed mice with *Ins1*Cre^Thor^ mice (Figure 3A; ref. 38). Levels of *Ywhaz* mRNA were significantly reduced in β14-3-3ζ–KO islets (Figure 3B), and a loss of 14-3-3ζ immunoreactivity was observed in β cells from β14-3-3ζ–KO mice (Figure 3C). In the absence of Cre recombinase, the presence of floxed alleles for *Ywhaz* did not significantly impact body weight, glucose tolerance, or insulin secretion in male or female mice (Supplemental Figure 2, A–F). No differences in i.p. glucose tolerance or insulin sensitivity were detected in β14-3-3ζ–KO mice (Figure 3, D and E). However, following an i.p. glucose bolus, significantly enhanced insulin secretion was observed in β14-3-3ζ–KO mice, and no differences in plasma glucagon levels were detected (Figure 3, F and G). The observed enhancement in insulin secretion in vivo was not observed in female β14-3-3ζ–KO mice (Supplemental Figure 2, G and H). No differences in β cell mass or islet size were observed between WT or β14-3-3ζ–KO mice (Figure 3, H and I), but β cell proliferation was significantly increased, as measured by PCNA^+^ or Ki-67^+^ β cells, was observed in β14-3-3ζ–KO mice (Figure 3, J and K). Deletion of 14-3-3ζ in β cells did not induce apoptosis (Figure 3L).

### Deletion of 14-3-3ζ in β cells leads to profound changes in the β cell transcriptome.

**Figure 4:**
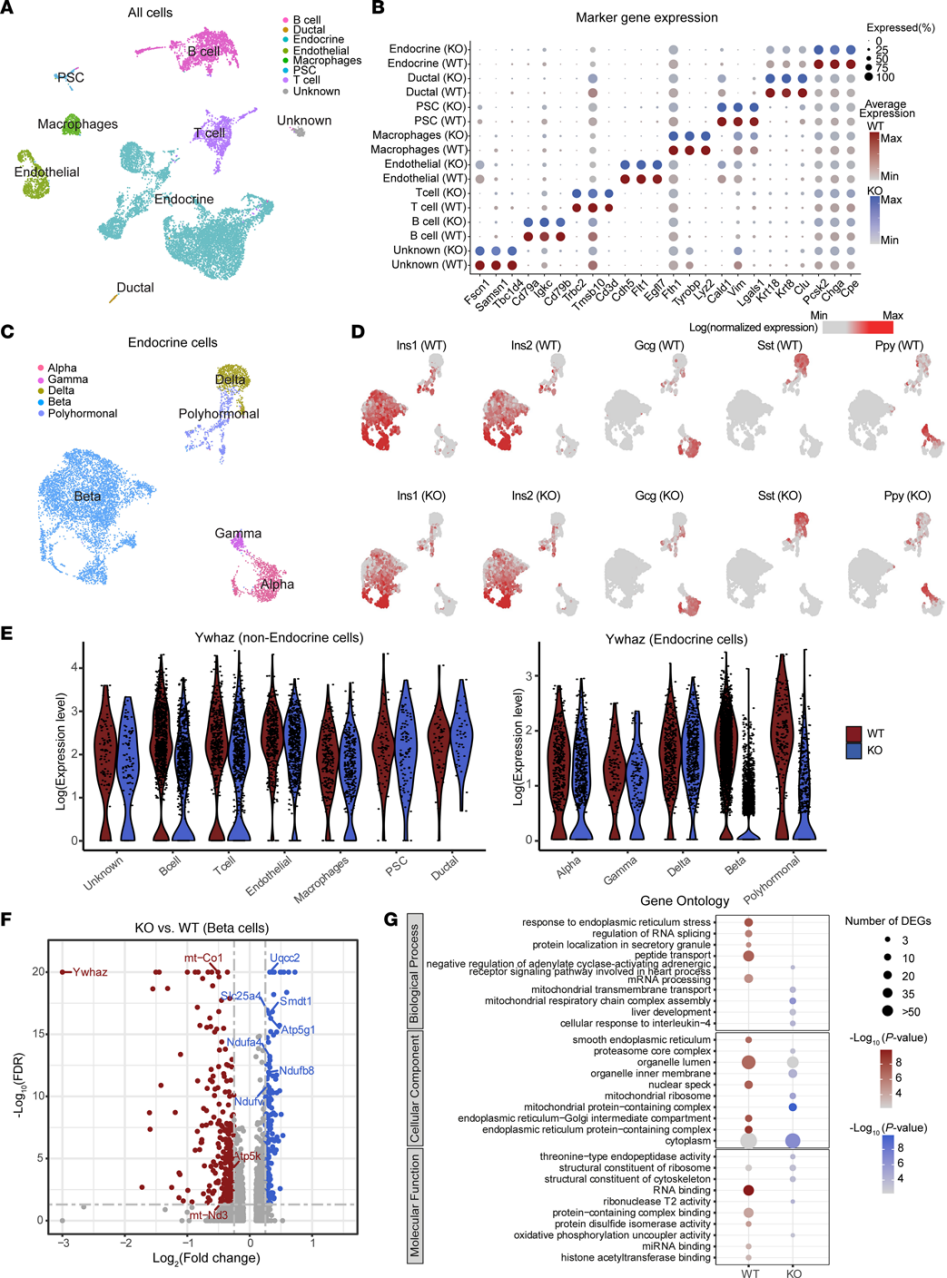
Single-cell transcriptome analyses of β14-3-3ζ–KO islets. (**A**) UMAP plot of all cells from WT and KO mouse pancreas colored by cell type. (**B**) Dot plots of candidate marker genes specific for cell types. The size and the color of the dot encode the percentage of cells and average expression level across all cells within each group (red and blue represent high expression levels in WT and KO cells, respectively). (**C**) UMAP plot of all endocrine cells from WT and KO mouse pancreas colored by cell subtypes. (**D**) UMAP plot as in **C** but split into WT and KO cells, illustrating the expression of the 4 endocrine hormones (both mouse Ins1 and Ins2 will encode for mature insulin protein): *Ins1*, *Ins2*, *Gcg*, *Sst*, and *Ppy*. The color scale is according to log-transformed normalization values, with light gray and red corresponding to the minimum and maximum expression, respectively. (**E**) Violin plot showing the expression of *Ywhaz* in different types of cells. (**F**) Volcano plot of the distribution of genes with at least 0.1 difference in log_2_ fold change in expression level, mapping the 351 upregulated genes (blue) and 358 downregulated genes (red). –Log_10_ FDR > 20 was set to avoid extremely high values. (**G**) Dot plot showing enriched GO from genes highly expressed in WT and KO β cells, respectively. The size and the color of the dot encode the numbers of DEGs and the significance of corresponding enriched GO. Data are an aggregate of *n =* 3 WT and *n =* 3 β14-3-3ζ–KO mice per group.

With the ability of 14-3-3ζ to regulate the subcellular localization of transcription factors (39–41), we next sought to understand if 14-3-3ζ deletion could impact the β cell transcriptome. Single-cell RNA-Seq was used to differentiate β cells from other endocrine cells within islets. Following quality control, we performed unbiased dimensional reduction analysis (Uniform Manifold Approximation and Projection [UMAP]) to identify cell populations (Supplemental Table 1), and we used known markers to manually assign each cluster a cell type identity and confirmed that deletion of 14-3-3ζ in β cells has no impact on key marker gene expression (Figure 4, A and B, and Supplemental Figure 3A). Any β cells derived from β14-3-3ζ–KO mice that had incomplete deletion of *Ywhaz* were removed for subsequent analysis. To further verify cell identity, we then integrated previously published work from a well-defined human islet data set (Supplemental Figure 3, C and D). Endocrine cells were identified by the expression of *Pcsk2* and *Chga* (Supplemental Figure 3B), and clustering of endocrine cells to their specific lineages was confirmed by the expression of *Ins1*, *Ins2*, *Gcg*, *Sst*, and *Ppy* (Figure 4, C and D). Across all identified cell types, deletion of *Ywhaz* was restricted to β cells, as no further reductions were detected in other cell types (Figure 4E and Supplemental Figure 4).

A total of 709 differentially expressed genes (DEGs) were identified in β cells of β14-3-3ζ–KO mice (FDR < 0.05, log_2_ fold change > 0.1, expressed in more than 15% of the cells). Of note was the discovery that, among the top upregulated DEGs in β14-3-3ζ–KO β cells were those involved in mitochondrial metabolism, such as *Uqcc2*, *Atp5g1*, and *Ndufa4* (Figure 4, F and G, and Supplemental Table 2). Additionally, *Smdt1*, which encodes the essential regulatory subunit of the Mitochondrial Uniporter Complex (MCU), was also found to be significantly upregulated (Figure 4F). The MCU is critical for regulating calcium uptake into the mitochondria and for GSIS (42). Gene ontology–based (GO-based) analysis of DEGs revealed that genes associated with regulation of RNA splicing (*P =* 2.7 × 10^–8^) were downregulated in β cells from β14-3-3ζ–KO mice, and this aligns with our previous finding implicating 14-3-3ζ in RNA processing and binding (10). Unexpectedly, enrichment of genes that participate in cell proliferation were not detected. Instead, deletion of 14-3-3 in β cells led to enrichment in genes associated with mitochondrial respiratory chain complex assembly (Biological Process) and mitochondrial protein complex (Cellular Component) (*P =* 4.3 × 10^–21^ and *P =* 3.8 × 10^–28^, respectively; Figure 4G, Supplemental Figure 3E, and Supplemental Table 3), which could account for the potentiation in GSIS observed in vivo (Figure 3F).

### 14-3-3ζ Regulates ATP-dependent insulin secretion.

**Figure 5:**
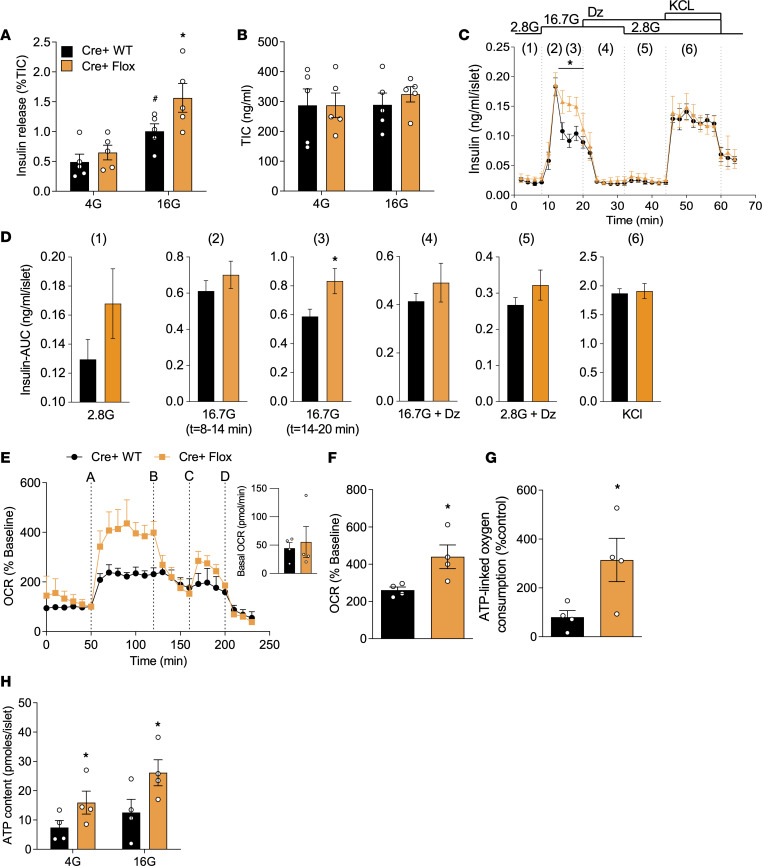
Enhanced β cell function ex vivo in β cell–specific 14-3-3ζ–KO mouse islets. (**A**) Isolated islets from Cre^+^ WT and Cre^+^ Flox mice were subjected to static glucose-stimulated insulin secretion assays. (**B**) Quantification of insulin content in acid-ethanol extracts from Cre^+^ WT and Cre^+^ Flox islets (*n =* 5 mice per group). (**C** and **D**) Perifusion of islets from 14-week-old Cre^+^ WT and Cre^+^ Flox mice was performed to examine insulin secretion dynamics in response to (lane 1) 2.8 mM glucose, (lanes 2 and 3) 16.7 mM glucose, (lane 4) 16.7 mM glucose + 200μM diazoxide, (lane 5) 2.8 mM glucose + 200μM diazoxide, or (lane 6) 35 mM KCl (**C**) and the corresponding AUCs (**D**) to each interval are shown (*n =* 3 mice per group). (**E**–**G**) Mitochondrial function, as determined by OCR (**E** and **F**) and ATP-linked oxygen consumption (**G**) were measured in Cre^+^ WT and Cre^+^ Flox islets. The inset image shows average basal OCR from 0 to 50 minutes. For OCR trace: (line A) glucose (16 mM); (line B) oligomycin (5 μM); (line C) FCCP (1 μM); and (line D) rotenone/antimycin (5 μM). (**H**) ATP content in islets from Cre^+^ WT and Cre^+^ Flox mice was quantified following exposure to different glucose concentrations (*n =* 4 per genotype) (*P < 0.05 when compared with Cre^+^ WT). Significance was determined by unpaired, 2-tailed Student’s *t* test (**C**, **D**, **F**, and **G**) or by 2-way ANOVA, followed by Tukey’s multiple-comparison tests (**A** and **H**).

Since deletion of 14-3-3ζ in β cells resulted in significant changes in the expression of genes associated with mitochondrial function and respiration (Figure 4, F and G), we next sought to determine whether 14-3-3ζ deletion would alter mitochondrial activity in β cells, which could explain the enhanced GSIS in β14-3-3ζ–KO mice (Figure 3F). Firstly, isolated islets from WT and β14-3-3ζ–KO mice were subjected to static GSIS assays, and under high-glucose conditions, significantly enhanced GSIS was detected from β14-3-3ζ–KO islets ex vivo (Figure 5A). No differences in insulin content were detected between groups (Figure 5B). When islets from WT and β14-3-3ζ–KO mice were subjected to perifusion, a potentiated second phase of insulin secretion was observed, which is consistent with the ATP-dependent phase of insulin release (Figure 5, C and D) (1, 2), and in the presence of diazoxide, the potentiated insulin secretory response was completely abrogated (Figure 5, C and D). Analysis of mitochondrial function and ATP synthesis revealed that 14-3-3ζ deletion in β cells could recapitulate the augmentation in mitochondrial function and ATP synthesis following acute pan–14-3-3 protein inhibition (Figure 5, E–H). Taken together, these findings suggest that, of the 7 mammalian isoforms, 14-3-3ζ is likely the key mammalian isoform in β cells that restrains insulin secretion and mitochondrial function.

In addition to the upregulation of genes associated the mitochondrial respiratory chain (Figure 4, F and G), we posited that the absence of 14-3-3ζ could relieve inhibitory effects on ATP synthase in mitochondria, similar to what has been observed in plants (29). This could result in further potentiated GSIS beyond an attained threshold. Interestingly, maximal insulin secretion in WT islets was reached at 16 mM glucose, and no further increase in insulin secretion was induced with 25 mM glucose (Supplemental Figure 1B). Although maximal GSIS from β14-3-3ζ–KO islets was also reached at 16 mM glucose, the magnitude of insulin release was significantly higher than in WT islets. Among the different concentrations of glucose, β14-3-3ζ–KO islets incubated in 10 mM glucose displayed elevations in mitochondrial activity and ATP synthesis that were equal to the maximal response of glucose (25 mM) in WT islets (Supplemental Figure 1, B–D). Collectively, these findings demonstrate that deletion of 14-3-3ζ in β cells has effects on regulating mitochondrial activity.

### Overexpression of 14-3-3ζ directly impairs insulin secretion and mitochondrial function.

We previously reported that transgenic overexpression of TAP–14-3-3ζ in mice was associated with defective insulin secretion in vivo, along with impaired glucose tolerance, but it was not clear if this decrease GSIS was islet specific (15, 43). To further explore the negative impact of 14-3-3ζ overexpression on insulin secretion and mitochondrial function, isolated islets from WT and TAP–14-3-3ζ mice were exposed to low and high glucose, and in contrast to what was observed with β14-3-3ζ–KO islets, Tandem Affinity Purification (TAP) islets demonstrated impaired GSIS with no detectable changes in total insulin content (Supplemental Figure 5, A and B). Moreover, mitochondrial function and total ATP synthesis were significantly decreased (Supplemental Figure 5, D–F). TAP-14-3-3ζ over-expression was also found to decreased *Ins1* mRNA levels (Supplemental Figure 5G). To confirm whether increased 14-3-3ζ function was responsible for the impairment in GSIS, islets from TAP mice were pretreated with 14-3-3i prior to exposure to high glucose exposure, and a restoration in GSIS comparable with WT mice was observed (Supplemental Figure 5C). Thus, overexpression of 14-3-3ζ has deleterious effects on GSIS from β cells.

### Deletion of 14-3-3ζ influence mitochondrial dynamics in the β cell.

**Figure 6:**
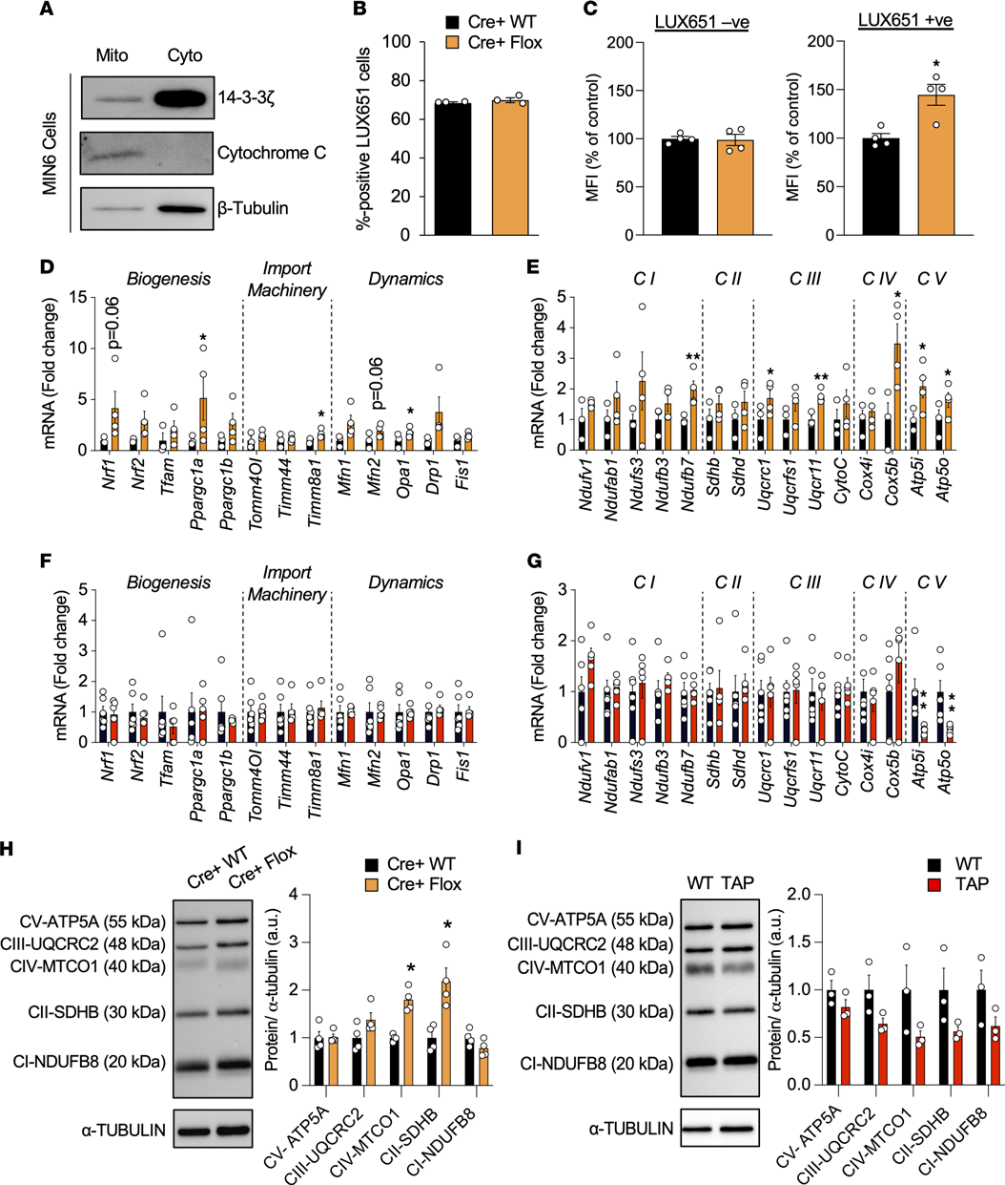
Detection of 14-3-3ζ in mitochondria, and analysis of its deletion, which leads to increases in mitochondrial mass and expression of genes associated with oxidative phosphorylation and biogenesis. (**A**) Mitochondrial (Mito) and cytoplasmic (Cyto) fractions were obtained from MIN6 insulinoma cells, resolved by SDS-PAGE, and probed for 14-3-3ζ. Cytochrome C and β-tubulin were used as mitochondrial and cytoplasmic loading controls, respectively (*n =* 3 independent experiments). (**B**) Cre^+^ WT and Cre^+^ Flox dispersed islet preparations were incubated with LUXendin-651 (LUX651; 400 nM) for 1 hour prior to detection by flow cytometry. The proportion of LUX651^+^ cells (LUX^+^/total cells counted) represents β cells from each preparation (*n =* 4 per group). (**C**) Dispersed β14-3-3ζ–KO islets were treated by MitoTracker green (100 nM) and LUXendin-651 (400 nM) to specifically label mitochondria and β cells, respectively. Histograms depict the median fluorescence intensity (MFI) of MitoTracker green in LUX651^–^ and LUX651^+^ cells (*n =* 4 per group; *P < 0.05 when compared with Cre^+^ WT). (**D**–**G**) Isolated mRNA from islets from Cre^+^ WT and Cre^+^ Flox mice (**D** and **E**) and WT and TAP mice (**F** and **G**) were subjected to qPCR analysis for mitochondrial biogenesis, import machinery, and dynamics genes (**D** and **F**), as well as for oxidative phosphorylation genes (**E** and **G**) (*n =* 3–4 mice per group; *P < 0.05; **P < 0.01 when compared with Cre^+^ WT or WT). (**H** and **I**) Western blot analysis of the OXPHOS mitochondrial complexes in islet extracts of Cre^+^ WT and Cre^+^ Flox mice (*n =* 3 per group; *P < 0.05 when compared with Cre^+^ WT mice) (**H**) or WT and TAP mice (*n =* 3 per group) (**I**). Significance was determined by unpaired, 2-tailed Student’s *t* test (**C**, **D**, **E**, **G**, and **H**).

Using affinity proteomics, we previously reported interactions between 14-3-3ζ and components of ATP synthase (10), and since ATP synthase is localized to the inner mitochondrial membrane (29, 44), it suggests that 14-3-3ζ could be mitochondrially localized. The localization of 14-3-3ζ within mitochondrial fractions has previously been reported in hippocampal neurons (45), but whether this occurs in β cells is not known. Purification of cytosolic and mitochondrial factors on MIN6 insulinoma cells revealed the presence of 14-3-3ζ within mitochondria (Figure 6A), suggestive of a localized function of 14-3-3ζ.

In HCT116 cancer cells, deletion of 14-3-3σ has been found to increase mitochondrial mass (46), demonstrating the possibility that 14-3-3ζ may similarly influence in β cell mitochondrial mass. As insulin secretory capacity positively correlates with mitochondrial mass (47), it suggests that changes in insulin secretion following 14-3-3ζ deletion could be associated with increased mitochondrial biogenesis. To measure mitochondrial mass in β cells, WT and β14-3-3ζ–KO islets were first incubated with the fluorescent probe, LUXendin-651 (LUX651), which enriches pancreatic β cells based upon GLP-1R expression (48). In dispersed WT and β14-3-3ζ–KO islet preparations, no differences in the proportion of LUX651^+^ cells, which represent β cells, were detected, and approximately 70% of counted cells were LUX651^+^, which aligns with the known proportion of β cells within a mouse islet (Figure 6B; ref. 49). The median fluorescence intensity (MFI) corresponding to MitoTracker Green was significantly higher in LUX651^+^ β cells from β14-3-3ζ–KO islets, demonstrating increased mitochondrial mass (Figure 6C). Notably, MitoTracker Green uptake is not dependent on mitochondrial potential. No differences in MFI were detected in LUX651^–^ cells between Cre^+^ WT and Cre^+^ Flox islets, indicating no impact on mitochondrial biogenesis in non-β cells (Figure 6C). Analysis of genes related to mitochondrial biogenesis and dynamics, such as *Ppgargc1a* and *Opa1,* were significantly increased in islets from β14-3-3ζ–KO mice, in addition to those regulating the mitochondrial import machinery (Figure 6D; ref. 47). In contrast, no significant differences in the expression of genes associated with mitochondrial biogenesis were observed in islets from TAP mice (Figure 6F).

Since 14-3-3ζ deletion increases mitochondrial function in β14-3-3ζ–KO islets and is also associated with enrichment of pathways associated with mitochondrial respiration within the β cell transcriptome (Figure 4G and Figure 5, E–H), we next examined if deletion of 14-3-3ζ in β cells could alter expression of mitochondrial complex proteins, which are necessary for ATP synthase (50). As measured by quantitative PCR (qPCR), various genes encoding complex proteins were significantly increased in β14-3-3ζ–KO islets (Figure 6E). This was in marked contrast to TAP islets, where only mRNA levels of *Atp5i* and *Atp5o* were decreased (Figure 6G). Protein abundance of Complex proteins were also found to be elevated in islets from β14-3-3ζ mice (Figure 6H), whereas no significant differences were observed in TAP islets (Figure 6I).

### β Cell dysfunction is associated with elevated 14-3-3ζ expression and can be alleviated by 14-3-3 protein inhibition.

**Figure 7:**
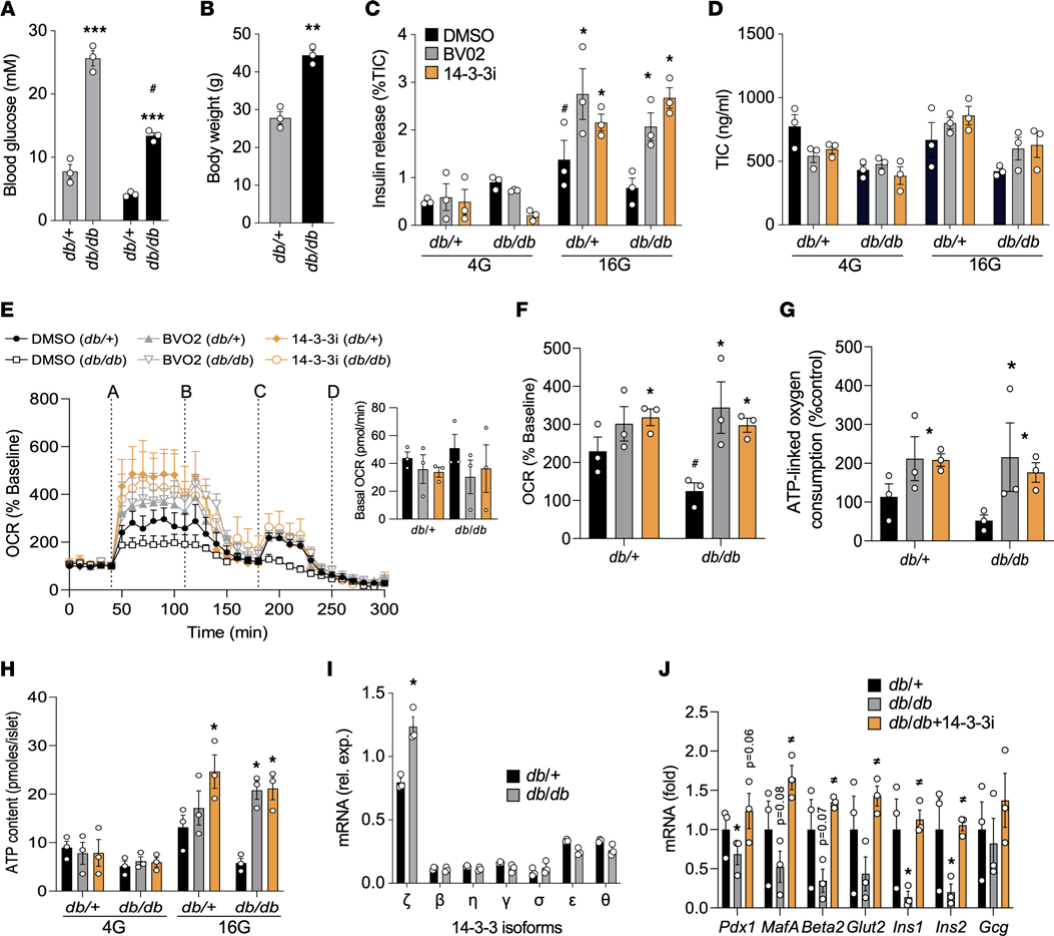
Potentiation of insulin secretion, mitochondrial function, and expression of mature β cell markers in islets from *db/db* mice. (**A** and **B**) Fed or fasted plasma blood glucose levels (**A**) and body weights (**B**) of 13-week-old *db/db* mice and control *db/+* mice (*n =* 3; **P < 0.01 and ***P < 0.0001 when compared with *db/+*; ^#^*P <* 0.05 when compared with fasting *db/+*). (**C** and **D**) Islets isolated from *db/db* and *db/+* were treated with pan–14-3-3 protein inhibitors (10 μM each) or DMSO, followed by static glucose-stimulated insulin secretion assays (**C**) or measurements of total insulin content (TIC) (*n =* 3 per group; ^#^*P <* 0.05 when compared with *db/+* 4G; *P < 0.05 when compared with 16G + DMSO). (**E**–**G**) Seahorse Extracellular Flux analysis to examine mitochondrial function, as determined by oxygen consumption (OCR, **E**, **F**) and ATP-linked oxygen consumption (**G**) rates. For OCR trace: (line A) glucose (16 mM); (line B) oligomycin (5 μM); (line C) FCCP (1 μM); and (line D) rotenone/antimycin (5μM) (*n =* 3 per group; *P < 0.05 when compared with DMSO). (**H**) ATP content of *db/db* and *db/+* mice islets treated with 14-3-3 inhibitors and quantified at different glucose concentrations. (**I** and **J**) Isolated mRNA from islets from *db/db* and *db/+* mice were subjected to qPCR analysis for 14-3-3 isoform expression (**I**) or *Pdx1*, *MafA*, *Beta2*, *Glut2*, *Ins1*, *Ins2*, and *Gcg* mRNA levels (**J**) (*P < 0.05 when compared with *db/+*; ^≠^*P <* 0.05 when compared with *db/db*). Significance was determined by unpaired, 2-tailed Student’s *t* test (**B** and **I**), 1-way ANOVA with Dunnett’s test (**J**), or 2-way ANOVA, followed by Tukey’s multiple-comparison tests (**A**, **C**, **F**, **G**, and **H**).

Due to the absence of circulating leptin, *db/db* mice develop significant obesity along with β cell dysfunction due to increased ER stress (51, 52). To examine if acute 14-3-3 protein inhibition could have beneficial effects by enhancing β cell function, islets were isolated from 13-week-old male control *db/+* and diabetic, obese *db/db* mice (Figure 7, A and B), and they were incubated with 14-3-3i or BV02. Similar to C57BL/6J mouse islets (Figure 1A), enhanced GSIS was observed from *db/+* and *db/db* mice acutely treated with 14-3-3i or BV02 for 2 hours (Figure 7C), and no effects on insulin content were observed (Figure 7D). Furthermore, 14-3-3 protein inhibition also resulted in enhanced mitochondrial function and ATP synthesis in *db/db* (Figure 7, E–H). To examine whether inhibition of 14-3-3 proteins could have similar effects in the context of obesity but without overt diabetes, islets from 9-week-old obese *ob/ob* and nonobese *ob/+* control mice (Supplemental Figure 6, A and B) were isolated and exposed to 14-3-3 protein inhibitors. Similar to *db/db* mice, enhanced GSIS and mitochondrial function were observed in *ob/ob* islets following 14-3-3 protein inhibition (Supplemental Figure 6, C–F).

Analysis of 14-3-3 isoform mRNA levels between *db/+* and *db/db* mice revealed significantly higher mRNA levels of *Ywhaz*, the gene encoding 14-3-3ζ (Figure 7I), further suggesting that elevated 14-3-3ζ expression impairs GSIS and mitochondrial function. In contrast, islets from *ob/ob* mice did not display differences in *Ywhaz* mRNA levels (Supplemental Figure 6G). β Cell dysfunction in *db/db* mice is associated is a loss of key genes associated with β cell identity (53), and when compared with *db/+* mouse islets, mRNA levels of *Ins1* and *Ins2* were significantly decreased in *db/db* islets, along with marked decreases in other mature β cell genes like *Pdx1*, *MafA*, and *Neurod1/Beta2* (Figure 7J). Interestingly, treatment of *db/db* islets with 14-3-3i for 72 hours was sufficient to restore expression of depressed genes to levels similar to nondiabetic *db/+* islets (Figure 7J).

**Figure 8:**
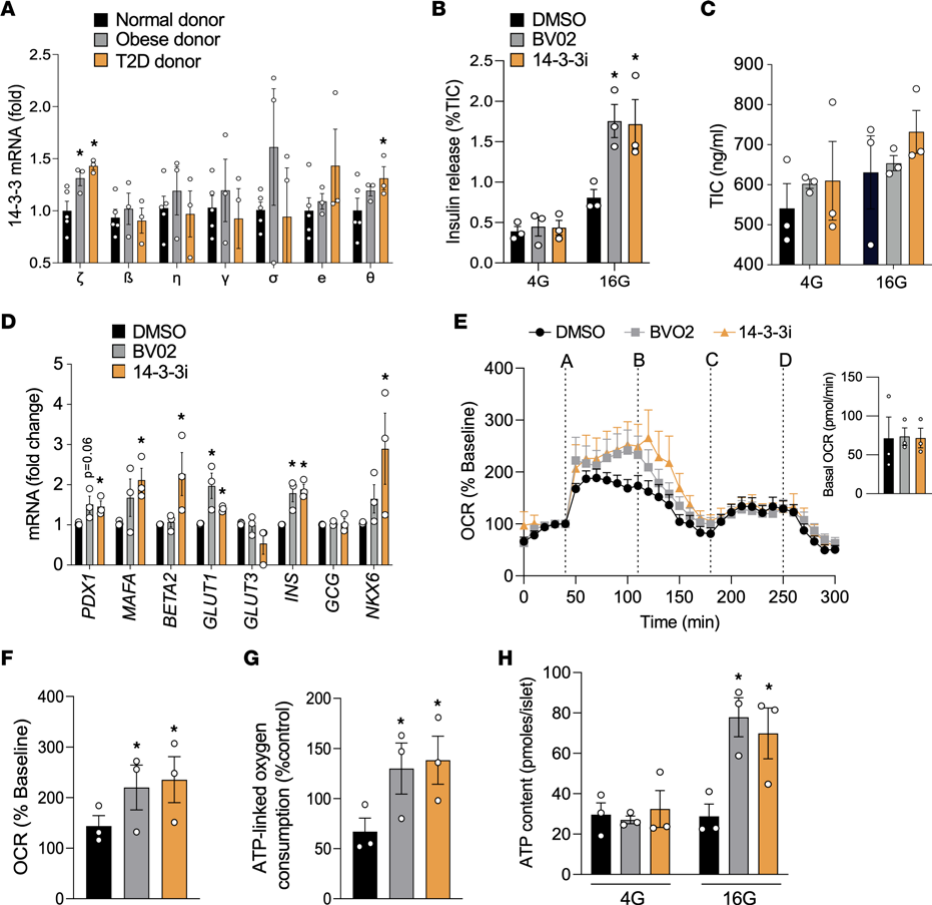
Inhibition of 14-3-3 proteins improves insulin secretory capacity and mitochondrial function in human islets from T2D donors. (**A**) Isolated mRNA from islets from normal, obese, and T2D human donors were subjected to qPCR analysis for 14-3-3 isoform expression (*n =* 3-5 donors per group; *P < 0.05 when compared with normal donor). (**B** and **C**) Human T2D islets (*n =* 3 donors) were treated with pan–14-3-3 inhibitors (10 μM each) for 2 hours, and a potentiation of glucose-stimulated insulin secretion was observed (**B**); no differences in total insulin content (TIC) were detected (**C**). (**D**) qPCR was used to measure changes in mRNA levels of *PDX1*, *MAFA*, *BETA2*, *GLUT1*, *GLUT3*, *INS*, *GCG*, and *NKX6* after 72 hours of treatment with 14-3-3 inhibitors (10 μM each). (**E**–**G**) T2D human islets were treated with pan–14-3-3 protein inhibitors (10 μM each) or DMSO followed by Seahorse Extracellular Flux analysis to examine mitochondrial function, as determined by OCR (**E** and **F**) and ATP-link oxygen consumption (**G**). For OCR trace: (line A) glucose (16 mM); (line B) oligomycin (5 μM); (line C) FCCP (1μM); and (line D) rotenone/antimycin (5μM). (**H**) ATP content in T2D human islets was measured after exposure to 14-3-3 inhibitors (10 μM each) and different glucose concentrations (*n =* 3 donors; *P < 0.05 when compared with DMSO). Significance was determined by 1-way ANOVA with Dunnett’s test (**A**, **D**, **F**, and **G**) or by 2-way ANOVA, followed by Tukey’s multiple-comparison tests (**B** and **H**).

Expression of *YWHAZ* mRNA in β cells and islets from cadaveric donor islets with type 2 diabetes is increased when compared with healthy donor islets (Figure 8A) (54). Moreover, *YWHAZ* mRNA levels were also significantly elevated in human islets from obese donors (Figure 8A). Prolonged exposure of human islets from T2D donors to 14-3-3i or BV02 for 72 hours stimulated the expression of genes associated with insulin biosynthesis (Figure 8D). Similar to human islets from healthy donors, pretreatment of islets from type 2 diabetic donors potentiated GSIS (Figure 2A versus Figure 8B), and 14-3-3 protein inhibition did not affect total insulin content (Figure 8C). Moreover, 14-3-3 protein inhibition enhanced mitochondrial function and ATP synthesis in type 2 diabetic human islets (Figure 8, E–H). Taken together, these findings demonstrate the beneficial effect of inhibiting 14-3-3 proteins to enhance β cell function in the context of overt diabetes.

## Discussion

Due to their ubiquitous expression, the importance of scaffold proteins belonging to the 14-3-3 protein family in glucose homeostasis and metabolism is often underappreciated. In the present study, we identify potentially novel regulatory roles of 14-3-3 proteins on GSIS from β cells, as they restrain insulin release due to inhibitory effects on mitochondrial function and the synthesis of ATP. We identify 14-3-3ζ as a key 14-3-3 protein isoform that is responsible for regulating mitochondrial mass and activity and, ultimately, insulin secretion from the murine β cell. Aside from effects on insulin release, inhibition of 14-3-3 proteins and deletion of 14-3-3ζ in β cells were also found to increase β cell proliferation. In models of β cell dysfunction,14-3-3 protein inhibition was able to improve β cell function and augment glucose-induced insulin release, in addition to increasing the expression of genes that regulate β cell identity and insulin biosynthesis. These findings highlight potentially new metabolic functions of 14-3-3ζ in β cells and suggest the possibility of targeting 14-3-3 proteins — specifically, 14-3-3ζ, to increase β cell function.

The signaling events underpinning GSIS have been well defined, and amplifying and nonamplifying pathways have been reported (55, 56). The key organelle that is central to GSIS is the mitochondrion, which is the primary site of ATP synthesis in the β cell. Generation of ATP requires the actions of Complex proteins (I–V) located in the inner mitochondrial membrane that work in concert to generate the proton gradient necessary for ATP synthesis (50). Complex V, or ATP synthase, is the key rate-limiting enzyme involved in ATP generation (29, 44), and inhibitory effects of 14-3-3 proteins on ATP synthase have been reported in chloroplasts and mitoplasts (29). Moreover, 14-3-3ζ deficiency in mouse platelets is associated with sustained intracellular ATP (30). To date, direct regulators of ATP synthase in β cells have not been established. Since we and others have detected 14-3-3ζ to be present within mitochondria under resting conditions (Figure 6A) (45) and to interact with ATP synthase (10), 14-3-3ζ is likely to exert a tonic inhibitory effect on ATP synthesis within mitochondria of β cells. This may explain how acute inhibition of 14-3-3ζ and its related isoforms in mouse and human islets with 14-3-3 protein inhibitors can significantly increase mitochondrial activity and ATP synthesis. Moreover, embryonic deletion of 14-3-3ζ in β cells caused changes to the β cell transcriptome such that genes associated with oxidative phosphorylation, mitochondrial respiration, and Complex proteins were significantly upregulated.

The loss of functional β cell mass, as defined by insulin secretory capacity and β cell number, is the key defining feature of diabetes (57, 58). We found that expression levels of 14-3-3ζ were inversely correlated with insulin secretory capacity and mitochondrial function, as β14-3-3ζ–KO islets and TAP–14-3-3ζ–overexpressing islets displayed potentiated or attenuated GSIS, respectively. Moreover, mRNA levels *Ywhaz/YWHAZ*, which encode for 14-3-3ζ, were significantly elevated in models of β cell dysfunction — namely, *db/db* islets and human islets from type 2 diabetic donors. Based on these results, it is tempting to speculate that targeting 14-3-3ζ in β cells could have positive effects on restoring insulin secretion and β cell mass. However, with the ubiquitous expression of 14-3-3 proteins, highly specific approaches to only target β cells still need to be developed. Notwithstanding, systemic inhibition of 14-3-3 proteins with FTY720 (Fingolimod), which was initially identified as a sphingosine-1-phosphate receptor agonist (59), has already been shown to ameliorate diabetes in models of type 1 and type 2 diabetes, and this highlights the potential of targeting 14-3-3 proteins. For example, FTY720 has been shown to delay or prevent diabetes progression in NOD mice due to effects on T cell function (60). Moreover, treatment of *db/db* mice with FTY720 reduced hyperglycemia by potently increasing β cell regeneration when administered for more than 5 months (61), and in spontaneously diabetic nonhuman primates, FTY720 administration was found to lower fasting glucose and improve β cell function (62). Alternatively, we and others have shown that it is possible to identify novel regulators of physiological pathways within the interactomes of 14-3-3ζ and its related isoforms (10–12), and this presents a different 14-3-3 protein–focused approach to uncover new therapeutic targets.

14-3-3ζ Does not bind directly to DNA or function as a transcription factor, but deletion of 14-3-3ζ led to changes in the β cell transcriptome. This effect is likely due to its ability to sequester transcription factors in the cytoplasm following their phosphorylation (39–41). Mitochondrial DNA–encoded (mtDNA-encoded) genes are distinct from nucleus-encoded genes and are primarily transcribed and translated within mitochondria, but cooperative pathways between these 2 compartments are needed for proper expression of these genes (63, 64). This can occur through interorganellar crosstalk between mitochondria and nuclei through shuttling of metabolites (63), or in the case of the present study, 14-3-3ζ could be influencing the subcellular localization of factors necessary for their expression. For example, Prohibitin-1 and Prohibitin-2 are nuclear-encoded proteins with known roles in mitochondrial biogenesis and function, and they regulate the expression of complex proteins (65, 66). Deletion of Prohibitin-2 in β cells has been shown to impair insulin secretion and mitochondrial function, resulting in a progressive worsening of glucose tolerance, and it promotes the development of diabetes (67). We previously identified interactions of 14-3-3ζ with Prohibitin-1 and Prohibitin-2 (10), and it is possible that deletion of 14-3-3ζ may lead to increased Prohibitin-1 and Prohibitin-2 abundance at mitochondria to exert positive effects on mitochondrial biogenesis or function. Lastly, as prohibitins have been shown to link mitochondrial function to cell proliferation (66, 68), this could account for the observed increases in cell proliferation in 14-3-3ζ–deficient β cells.

Genetic inactivation of all 14-3-3 proteins has been shown to induce premature cell cycle entry and increase proliferation (31), and future examination of how 14-3-3ζ regulates β cell proliferation will be of interest. Based on previous studies, 14-3-3ζ could influence proliferation by regulating the localization and function of cyclins and cyclin-dependent kinases (CDKs) (13, 31, 69–72). It is also possible that 14-3-3ζ may influence the activity of other kinases — such as DYRK1A — that regulate β cell proliferation (35, 73). Interestingly, the kinase activity of DYRK1A is positively influenced by interactions between DYRK1A and 14-3-3 proteins (70, 71). In the present study, we found that deletion of 14-3-3ζ in β cells leads to increases in the expression of PCNA or Ki-67, which are markers of proliferation; however, no increases in β cell mass were detected. It is unclear why β cell mass was not increased, despite increased expression of proliferative markers, and it suggests a failure or block in the completion of a cell cycle. Further work is required to explore this in detail.

In summary, this study reports potentially novel, physiological roles of 14-3-3 proteins in pancreatic β cells and highlights the possibility of targeted inhibition of 14-3-3 proteins — or specifically 14-3-3ζ — to enhance insulin secretion. Inhibiting 14-3-3 protein function and deletion of 14-3-3ζ in β cells had profound beneficial effects on mitochondrial function, glucose-stimulated insulin release, and β cell proliferation. In contrast, increased 14-3-3ζ expression was found to be inversely associated with GSIS, as islets from diabetic *db/db* mice or human islets from donors with type 2 diabetes displayed higher levels of *Ywhaz/YWHAZ*. Moreover, inhibition of 14-3-3 proteins in these diabetic models was sufficient to enhance insulin secretion and mitochondrial function. Overall, results from the present study reveal roles of the 14-3-3 protein family in pancreatic β cells and deepen our understanding of the regulation of glucose-stimulated insulin release.

## Methods

### Animals.

β Cell–specific 14-3-3ζ–KO mice on a C57BL/6J background and TAP–14-3-3ζ transgenic mice on a CD1 background were housed on a 12-hour light/dark cycle with free access to water and standard rodent chow diet (15% fat by energy, Teklad). The β14-3-3ζ–KO mice were generated by breeding *Ins1*Cre^Thor^ mice (The Jackson Laboratory, 026801) (38) with mice harboring alleles with LoxP sites flanking exon 4 of *Ywhaz*, the gene encoding 14-3-3ζ (Toronto Center for Phenogenomics) (74, 75). TAP–14-3-3ζ mice express TAP-tag consisting of protein A and calmodulin-binding peptide, separated by a TEV protease cleavage site, under the control of the human UbiC promoter (43). Littermate controls (Cre^+^ WT for β14-3-3ζ KO and WT for TAP–14-3-3ζ) were used in all experiments. For glucose- and insulin-tolerance tests, β14-3-3ζ–KO or TAP–14-3-3ζ mice were fasted for 6 or 4 hours, respectively, followed by i.p. injection of 2 g/kg glucose or 0.75U/kg Humulin R insulin (Eli Lilly). Blood glucose levels were measured with a glucometer (Contour Next, Ascensia Diabetes Care). Plasma insulin and glucagon were measured by ELISA (Alpco). Male and female C57BL/KsJ *db/db* (stock no. 000697) and C57BL/6J *ob/ob* (stock no. 000632) mice and age-matched, lean control mice (C57BL/KsJ *db/+* or C57BL/6J *ob/+*, respectively) were purchased from The Jackson Laboratory.

### Mouse and human islets.

Pancreatic islets were isolated by collagenase (type XI; Sigma-Aldrich) digestion of total pancreas, as previously described (56). Isolated islets were handpicked under a stereoscope and cultured overnight at 37°C in 11.1 mM glucose RPMI 1640 medium with sodium bicarbonate, supplemented with 10% FBS, 10 mM HEPES (pH 7.4), 2 mM L-glutamine, 1 mM sodium pyruvate, 100 U/mL penicillin, and 100 μg/mL streptomycin before the start of the experiments (all cell culture reagents from Thermo Fisher Scientific).

Human islets (75%–90% pure; 13 different donors without any known disease and 3 type 2 diabetic donors) were from the Alberta Diabetes Institute IsletCore (Edmonton, Alberta, Canada) and the Integrated Islet Distribution Program (IIDP; City of Hope, Duarte, California, USA). Isolated human islets were handpicked and cultured overnight in 5 mM glucose DMEM, supplemented with 10% FBS and 1% penicillin/streptomycin before the start of the experiments. Donor characteristics can be found in Supplemental Table 4.

### Insulin secretion and ATP content measurement.

Mouse islets were transferred to RPMI 1640 medium with 4 mM glucose and human islets to DMEM with 5 mM glucose for 2 hours to achieve baseline insulin secretion. Batches of 10 islets for insulin secretion and 80 islets for ATP content were washed and preincubated for 45 minutes in Krebs Ringer buffer-Hepes (KRBH) at pH 7.4 containing 4 mM glucose and 0.5% defatted BSA, as well as various pharmacological agents, or DMSO, followed by incubation for 60 minutes in KRBH with different concentrations of glucose in the presence or absence of pharmacological agents.

Islet perifusion (Biorep Perifusion System V5; Biorep Technologies Inc, Miami Lakes, FL, USA) was performed to measure insulin secretion from 80 mouse islets of equal size for each genotype. Islets were perifused at 37°C and a rate of 0.1 mL per minute with KRBH, pH 7.4, plus 0.5% BSA and glucose, diazoxide, and KCl, as indicated. After 20 minutes preperifusion in 2.8 mM glucose, the perifusate was collected every 2 minutes for 6 minutes in 2.8 mM glucose, 16 minutes in 16.7 mM glucose, 12 minutes in 16.7 mM plus 200μM diazoxide, 12 minutes in 2.8mM glucose plus 200μM diazoxide, 16 minutes in 2.8 mM glucose plus 200μM diazoxide plus 35 mM KCL, and finally 6 minutes in 2.8 mM glucose. Total insulin released into medium, perifusate, and total insulin content (TIC) extracted by acid ethanol was determined by radioimmunoassay (MilliporeSigma).

Islet ATP content was determined by ATP bioluminescent assay kit (Sigma-Aldrich), as described before (76). Briefly, human and mouse islets were sonicated for 1 min in 400 μL of PBS on ice. To measure the amount of ATP via luminescence, an isotonic solution containing luciferase-luciferin (LL) prepared from a vial of ATP assay mix powder (Sigma-Aldrich) was used. This isotonic LL was mixed with KRBH, and ATP-dependent LL bioluminescence was measured by a TD-20/20 luminometer (Turner Designs). Data were recorded via Spreadsheet Interface Software v.1.2.0 (Turner BioSystems Inc.).

### Oxygen consumption and mitochondrial function.

Oxygen consumption was measured at 37°C from isolated mouse and human islets after overnight recovery using a Seahorse XF24 analyzer (Agilent). Islets were seeded at a density of 75 islets/well. After basal respiration measurement for 40 minutes, glucose levels were elevated to 16 mM, followed by 3 successive injections of 5 μM oligomycin, 1 μM FCCP, 5 μM rotenone, and 5 μM antimycin (Sigma-Aldrich) to assess uncoupled respiration, maximal mitochondrial respiration, and nonmitochondrial respiration, respectively. ATP production was calculated by measuring the decrease in OCR upon injection of oligomycin (56).

### Islet proliferation.

Isolated mouse islets were dispersed in trypsin (0.05%) for 5 minutes at 37°C. To image proliferative β cells, dispersed islets were seeded in chamber slides (Thermo Fisher Scientific) coated with poly-D-lysine hydrobromide (Sigma-Aldrich), and cells were cultured in RPMI 1640 medium containing 11 mM glucose with 10% FBS for 72 hours in the presence of 10 μM of BVO2, 14-3-3i, and harmine (Sigma-Aldrich). Media were changed every 24 hours. After successive washes with PBS, cells were fixed with 4% paraformaldehyde, followed by coimmunostaining for insulin (ab63820; 1:50; Abcam) and the proliferative markers Ki-67, (ab15580; 1:300; Abcam).

Human islets were handpicked, washed with PBS, and dispersed in trypsin 0.05% (Thermo Fisher Scientific) for 5 minutes at 37°C. At the end of the digestion, cells were washed, resuspended, and plated in chamber slides pretreated with poly-D-lysine hydrobromide. After overnight incubation, dispersed human islets were incubated in DMEM with 1% FBS for 72 hours in the presence of 5 mM glucose, 10 μM BVO2, 14-3-3i, and harmine. The medium was changed every 24 hours. At the end of treatment, cells were fixed and immunostained for insulin and Ki-67. Following incubation with insulin and Ki-67 antibodies, Alexa Fluor 594– and Alexa Fluor 488–conjugated secondary antibodies were used, respectively (1:500 dilution; Jackson ImmunoResearch). Slides were coverslipped after addition of ProLong Gold mounting medium (Thermo Fisher Scientific). All images were taken with an Evos FL fluorescence microscope (Thermo Fisher Scientific). Proliferation was calculated as the percentage of Ki-67^+^ and insulin^+^ cells over the total insulin^+^ cell population. At least 1500 β cells were manually counted per condition.

### Flow cytometry.

β Cell proliferation was also measured by flow cytometry. After treating with 14-3-3i or harmine for 72 hours, islets were dispersed and dead cells were labeled using the LIVE/DEAD Fixable Aqua (405 nm) Dead Cell Stain Kit (BD Biosciences). EdU detection, using the Click-iT Plus EdU Flow Cytometry Assay Kit with Alexa Fluor 488, and immunostaining were performed according to the manufacturer’s instructions (Thermo Fisher Scientific). Insulin was detected using the primary fluorophore-coupled antibody Alexa Fluor Mouse Anti-Insulin (BD Biosciences, catalog 565689, dilution 1:50). Flow cytometry analysis was performed using a LSRIIB flow cytometer with BD FACSDiva software (BD Biosciences). Dead cell stain, EdU-labeled, and insulin-labeled cells were detected using the 405, 488, and 640 nm lasers coupled with 525/50, 530/30, 670/14 nm BP filters, respectively. Proliferation was calculated as the percentage of double-positive cells for EdU and insulin over the total insulin^+^ cell population.

To measure mitochondrial mass, 150 mouse islets from 10-week-old Cre^+^ WT or Cre^+^ Flox mice were washed PBS containing 2 mM EDTA, followed by dispersion with trypsin at 37°C for 5 minutes, and passed through a 30 gauge needle. Islet cells were then rinsed with PBS twice and incubated with 50 nM FITC-conjugated MitoTracker green (Thermo Fisher Scientific) for 30 minutes at room temperature, followed by coincubation with 400 nM LUXendin-651 (48) for an additional 30 minutes. Labeled cells were monitored for FITC (488 nm/530 nm) and APC (633 nm/780 nm) using flow cytometry (LSR-II, BD Biosciences) and FACSDiva software (BD Biosciences). Data were analyzed using FlowJo v10.7. Mean fluorescent intensity (MFI) was calculated for FITC-MitoTracker in the LUXendin-651^–^ (FITC-MitoTracker^+^/LUXendin-65^–^, Q1) and LUXendin-651^+^ (FITC-MitoTracker^+^/LUXendin-651^+^, Q2) populations and compared between Cre^+^ WT and Cre^+^ Flox islets. Gating strategies used to measure EdU^+^ cells, and MitoTracker green measurements of mitochondrial mass can be seen in Supplemental Figure 7.

### IHC, β cell mass measurement, and TUNEL assay.

Whole pancreata were removed from WT and β14-3-3ζ–KO mice, weighed, fixed in 4% paraformaldehyde, embedded in paraffin, and sectioned to 6 μm thickness. A minimum of 3 sections, 72 μm apart, were used in all studies. Antigen retrieval at 95°C was performed using EZ-Retriever System (Biogenex) with 10 mM sodium citrate buffer. β Cell proliferation was assessed, as described above. The In Situ Cell Death Detection Kit (TUNEL; Roche Applied Sciences) was used to measure β cell apoptosis (Roche; ref. 15). To measure β cell mass in pancreatic sections, IHC was performed with an insulin antibody (1:200 dilution; Ab 3014, Cell Signaling Technology) and the SignalStain DAB Substrate Kit (Cell Signaling Technology). Hematoxylin was used for counterstaining. Slides were monitored using a high-resolution scanner (Aperio ImageScope 12.3.3) to assess the areas of insulin^+^ β cells and the whole pancreas, followed by calculating β cell mass (total β cell area/total pancreas weight).

### MIN6 cell culture and mitochondrial fractionation.

Mouse insulinoma 6 (MIN6) cells (a gift from Jun-ichi Miyazaki, Osaka University, Suita, Japan) (passages 19–23) were cultured at 25 mM glucose in DMEM supplemented with 10% FBS and 1% penicillin/streptomycin at 37°C in a humidified atmosphere (5% CO_2_, 95% air; ref. 77). Cells were seeded in a 75 cm^2^ culture flask for 5–7 days to reach a 70%–80% confluence at the day of the experiment. Purification of cytoplasmic and mitochondrial fractions or pure mitochondria were done from 20 × 10^6^ cells (Mitochondria Isolation Kit for Cultured Cells, Thermo Fisher Scientific).

### Single-cell RNA-Seq of pancreatic islets.

Islets isolated from WT and β14-3-3 KO mice at 12–14 weeks were dispersed after an overnight recovery as described above. Dead cells were removed by using a dead cell removal kit and MS columns (Miltenyi Biotec GmbH). Single-cell RNA-Seq libraries were made with the 10× Genomics Chromium Next GEM Single Cell 3′ Library Kit (v3.1) and sequenced using an Illumina NovaSeq6000 (100 bp × 2, 50,000 reads/cell). Approximately 9600 cells were loaded per sample with an anticipated recovery of 6000 cells. Information related to read mapping and gene expression quantification, quality control and normalization, data integration and clustering, and marker gene expression can be found in Supplemental Methods. All raw data files are available in the Gene Expression Omnibus (GEO; GSE186529; https://www.ncbi.nlm.nih.gov/geo/query/acc.cgi?acc=GSE186529).

### Statistics.

Statistical analyses were performed through GraphPad Prism 9 by using Student’s *t* test or ANOVA, followed by Dunnett, Tukey’s, or Bonferroni post hoc tests. Statistical significance is indicated in the figures as follows: *P < 0.05; **P < 0.01; ***P < 0.001. All data are presented as mean ± SEM.

### Study approval.

All procedures were approved by the institutional committee for the protection of animals (Comité Institutionnel de Protection des Animaux du Centre Hospitalier de l’Université de Montréal, protocol CM20043GLs). Ethical approval for the use of human islets was obtained from the Institutional Ethics Committee of the Centre Hospitalier de l’Université de Montréal (protocol 18-726 CER).

## Author contributions

YM designed and performed experiments, analyzed data, and wrote and edited the manuscript. GEL designed experiments and wrote and edited the manuscript. JJT, AG, SAC, FP, SSP, MG, and EF performed experiments. CZ analyzed data and provided bioinformatic analysis. JA and JB provided reagents. EEM, SP, and DJH edited the manuscript and provided reagents. GEL is the guarantor of this work.

## Supplementary Material

Supplemental data

Supplemental table 1

Supplemental table 2

Supplemental table 3

Supplemental table 4

Supplemental table 5

## Version 1 03/17/2022

In-Press Preview

## Version 2 04/22/2022

Electronic publication
