# Supplementary material for: 14-3-3ζ Constrains insulin secretion by regulating mitochondrial function in pancreatic β cells
Source: JCI Insight. 2022 Apr 22;7(8):e156378. doi: 10.1172/jci.insight.156378 (PMC9089799; doi:10.1172/jci.insight.156378)
Supplement: Supplemental data [file jciinsight-7-156378-s078.pdf]

Supplemental Information- Methods

Quantitative Real-Time PCR

Total RNA was extracted from islet cells with the RNeasy Micro Kit (Qiagen, Hilden, Germany) and first-strand cDNA was synthesized from 0.5 to 1µg of total RNA with the High-Capacity cDNA Reverse Transcription Kit (ThermoFisher Scientific, Waltham, MA). Quantitative real-time PCR (qPCR) was performed with SYBR Green chemistry using the QuantStudio 6-flex Real-time PCR System (Thermo Fisher Scientific). Expression levels of mouse and human genes were normalized to *Actinb* or *cyclophilin* or *ACTINB*, respectively, by the ΔC_t method. Primer sequences are listed in Supplemental Table 5.

Immunoblotting

Cells were lysed in RIPA buffer, supplemented with protease and phosphatase inhibitors, as previously described (10, 13). Proteins were resolved by SDS-PAGE, transferred to PVDF membranes, and probed with antibodies against 14-3-3ζ (Ab 9639), α-tubulin (Ab 2144), β-tubulin (Ab 86298), β-actin (Ab 3700) (1:1000 dilution; Cell Signaling Technology, Danvers, MA), OXPHOS complexes (110413; 1:250 dilution; Abcam, Toronto, Canada) and Cytochrome C (556432, 1:1000 dilution; BD Pharmingen, San Diego, CA, USA).

Single-cell RNA-seq of pancreatic islets

Reads mapping and gene expression quantification

Sequencing data were aligned and quantified by using the Cell Ranger Pipeline v6.0.2 (10x Genomics) with the default setting against the mm10 reference genome (v.2020-, downloaded from the 10X Genomics website).

Quality control and normalization

We used 3 parameters to assess the quality of our cells. First, cut-off based on the number of expressed genes (nGene) and the expression level of mitochondrial genes (percent.mito) were used to filter out the low-quality cells. In detail, cells with percent.mito less than 0.15 and 1500 < nGene < 6000

were retained. In addition, as previously reported, reads belonging to lincRNAs Gm42418 and AY036118 were assigned toward the expression of Rn45s repeat (82). Cells with more than 5% of total reads mapped to Rn45s repeat are likely contaminated or dead cells (83), as such, those cells were further removed from our analysis. Together these stringent parameters were important and included in our study considering that mitochondrial activity is impacted by 14-3-3ζ protein. Following the selection of high-quality cells, we then further filtered based on genes. Genes were considered truly expressed if they contained one or more counts in at least five cells (assessed for each sample separately). Log-normalized counts were calculated using the deconvolution strategy implemented by the computeSumFactors function in scran package (v.1.14.6) (84). We then performed rescaled normalization using the multiBatchNormfunction in the batchelor package (v.1.2.4) so that the size factors were comparable across samples (85). The log-normalized expression after rescaling was used in marker gene detection and differential gene expression analysis.

Data integration, dimensionality reduction and clustering

We integrated the filtered count matrices from the wildtype and knockout samples using the sctransform approach implemented in Seurat package (86, 87) on the 3000 anchor features. After integration, principal component analysis (PCA) was performed on the integrated data followed by embedding into low dimensional space with Uniform Manifold Approximation and Projection (UMAP) based on the top 30 dimensions. Formed clusters generated by FindClusters function from Seurat package were assigned to cell types by consulting the expression of known marker genes and automatic annotation from SingleR package (v1.4.1) (88). To obtain the different types of endocrine cells present, cells from the endocrine cluster were further extracted and integrated based on 2000 anchor features and top 30 dimensions. In addition, scRNA-sequencing data of human pancreatic islets was integrated using the same parameters as above, with our dataset to further confirm the cell identities (56). Ortholog genes with the same gene symbols according to Ensemble annotation were used for the integration.

Marker gene detection and differential gene expression analysis

Marker genes for each cell type were identified by FindAllMarkers function using ‘roc’ test from Seurat package. Top 50 Marker genes conserved in wild-type and mutant with at least average power more than 0.6 were selected (sup.marker.genes.tsv). Differential gene expression analysis between wild-type and knockout was performed using ‘MAST’ test implemented in FindMarkers function from Seurat package (89). Genes with an FDR less than 0.05, log_2-(fold change) more than 0.1, and expressed in more than 15% of the cells were considered differentially expressed. The Gene Ontology (GO) enrichment analysis for differentially expressed genes was conducted using the TopGO package (90). Adrian Alexi’s improved weighted scoring algorithm and Fisher’s test were used to define the significance of GO term enrichment. Functional enrichment analysis was performed using the ClusterProfile package (91). KEGG functional annotations were downloaded from EnrichR database (92). Significantly enriched GO and functional terms were identified as those with a *p*-value and FDR less than 0.05, respectively. β-cells from knockout mice with expressed *Ywhaz* expression were excluded from differential gene expression analysis.

Supplemental Figure Legends

Supplemental Figure 1- Pan-inhibition of 14-3-3 proteins or deletion of 14-3-3ζ potentiate insulin secretion and mitochondrial respiration in a glucose-dose dependent manner. (A) Effect of pan 14-3-3 protein inhibition on glucose-stimulated insulin secretion in mouse islets incubated at 4, 10, 16 and 25 mM glucose for 1hr (n = 3, *p < 0.05; **p <0.01 when compared to 4G; # p <0.05 when compared to DMSO). **(B-D)** Effect of β-cell-specific deletion of 14-3-3ζ on glucose-stimulated insulin secretion (B), OCR (C) and ATP synthesis (D) in islets incubated at 4, 10, 16 and 25 mM glucose for 1hr (n = 3 per group), *p <0.05 when compared to Cre^{+} WT).

Supplemental Figure 2- β-cell Cre expression does not affect body weight, glucose tolerance, and glucose-stimulated insulin secretion in male and female mice. (A-C) No differences in body weight (A) due to Cre expression were detected in male mice. Expression of Cre in β-cells did not influence glucose tolerance (B) or insulin secretion (C) following an intraperitoneal glucose (2 g/kg) bolus (n= 4-7 per group). **(D-F)** In female mice, no differences in body weight (D), glucose tolerance, (E), or insulin sensitivity (F) were detected due to the expression of Cre in β-cells. **(G,H)** No differences in glucose-stimulated insulin secretion following *i.p* glucose (2g/kg) in Cre^{-} (G) and Cre^{+} (H) female mice (n=3-7 mice per group).

Supplemental Figure 3- Clustering of islet cells according to established markers.
(A) UMAP plot as in Figure 3A depicting individual samples. **(B)** Expression of endocrine marker genes (*Pcsk2* and *Chga*) overlaid onto the UMAP plot as shown in Figure 3A. **(C, D)** UMAP plot of the integrated dataset of all cells (C) and endocrine cells (D) from human pancreas dataset colored by cell types. **(E)** Dot plot showing enriched KEGG pathways from genes highly expressed in wild-type and knockout beta cells, respectively.

Supplemental Figure 4- No differences in *Ywhaz* mRNA levels were observed in non β-cells from islets of β14-3-3ζKO mice. Levels of *Ywhaz* mRNA were determined from identified cell types from single-cell RNA-seq analysis of Cre+WT and Cre+Flox islets (n=3 mice per group). Data correspond to Figure 4.

Supplemental Figure 5- Over-expression of 14-3-3ζ in β-cells inhibits glucose-stimulated insulin secretion and mitochondrial function. (A,B) Islets isolated from 14-3-3ζ TAP transgenic mice and littermate WT controls were subjected to static glucose-stimulated insulin secretion assays (A). Quantification of insulin content (B) in acid-ethanol extracts from WT and 14-3-3ζTAP islets (n= 9 mice per group, *p < 0.05 when compared to WT). **(C)** Impaired GSIS due to 14-3-3ζ over-expression can be restored by pan-14-3-3 protein inhibition (*:p< 0.05 when compared to DMSO WT; # :p< 0.05 when compared to DMSO TAP). **(D, E)** Seahorse Extracellular Flux analysis to examine mitochondrial function, as determined by oxygen consumption rate (D, OCR) and ATP synthesis rates (E) (*: p< 0.05 when compared to WT). **(F)** ATP content of WT and TAP mice islets quantified at different glucose concentrations. **(G)** Isolated mRNA from islets from WT and TAP mice were subjected to qPCR analysis for *Pdx1, Gcg, Ins2, Ins1, MafA, Sst and Ywhaz* expression (n = 3 per group; *:p<0.05 when compared to WT).

Supplemental Figure 6- Effects of 14-3-3 protein inhibition on insulin secretion, mitochondrial function. and gene expression in islets of *ob/ob* mice. (A, B) Blood glucose (A) and body weights (B) of 9-week-old *ob/ob* mice and their control *ob/+* fed or fasted for 16h (n= 3; **: p <0.01; ***p <0.0001 when compared to *ob/+*). **(C-E)** Islets isolated from *ob/ob* and *ob/+* were treated with pan-14-3-3 protein inhibitors (10μM each) and subjected to static glucose-stimulated insulin secretion assays (C) and Seahorse Extracellular Flux analysis to examine mitochondrial function, as determined by OCR (D) and ATP synthesis rates (E) (*p < 0.05 when compared to DMSO). **(F)** ATP content of *ob/ob* and *ob/+* mice islets treated with 14-3-3 inhibitors and quantified at different glucose concentrations, (*p < 0.05; ***p < 0.001 when compared to DMSO). **(G)** Isolated mRNA from islets from *ob/ob* and *ob/+* mice were subjected to qPCR analysis for 14-3-3 isoform expression.

Supplemental Figure 7- Gating strategies used for Flow Cytometry. Gating strategies for flow cytometric analyses of proliferating (Insulin-positive, EdU-postive) β-cells **(A)** or measurements of mitochondrial mass (Mitotracker green, Luxendin 651+) in islet cells **(B)**.

Supplemental Tables/ Spreadsheets

Supp. Table 1- Number of cells counted per biological replicate

Supp. Table 2- Differentially Expressed Genes

Supp. Table 3- Gene ontology

Supp. Table 4- Donor characteristics

Supp. Table 5- Primers

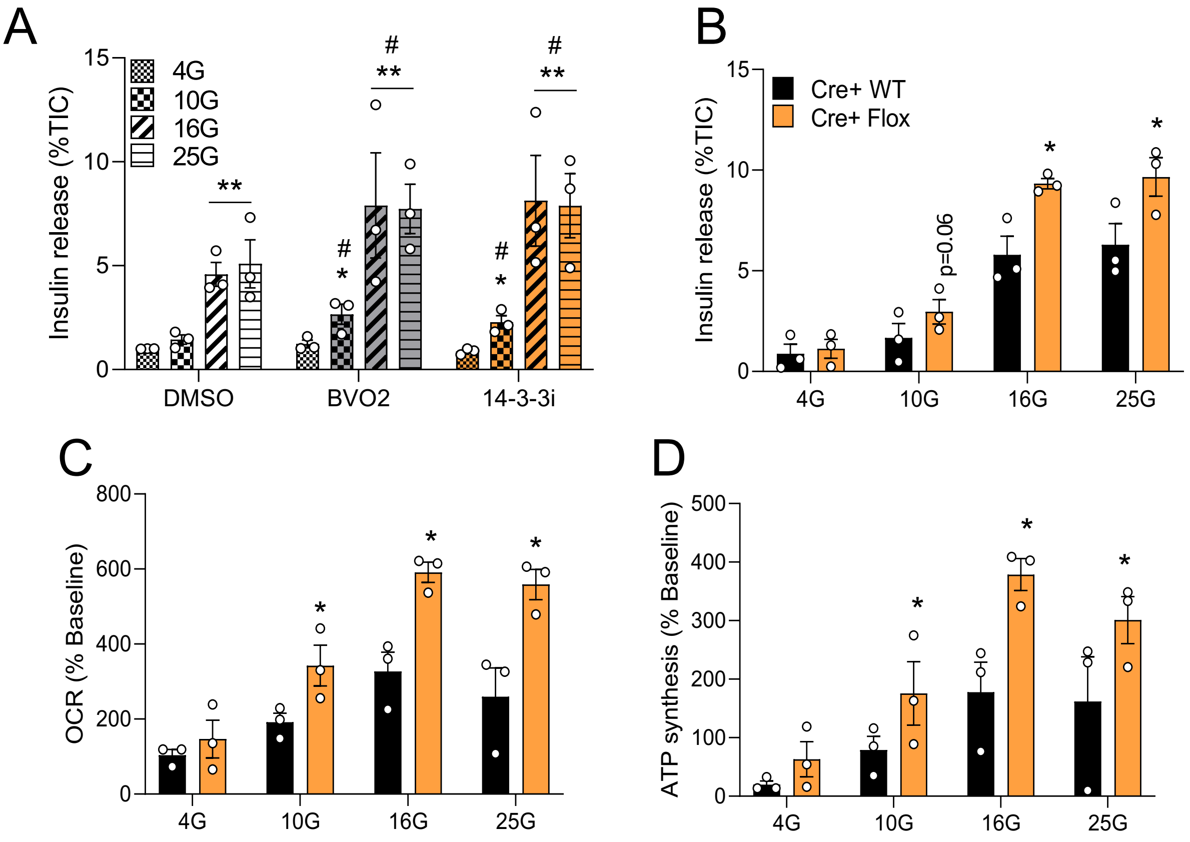

Supplemental Figure 1. Pan-inhibition of 14-3-3 proteins or deletion of 14-3-3ζ potentiate insulin secretion and mitochondrial respiration in a glucose-dose dependent manner

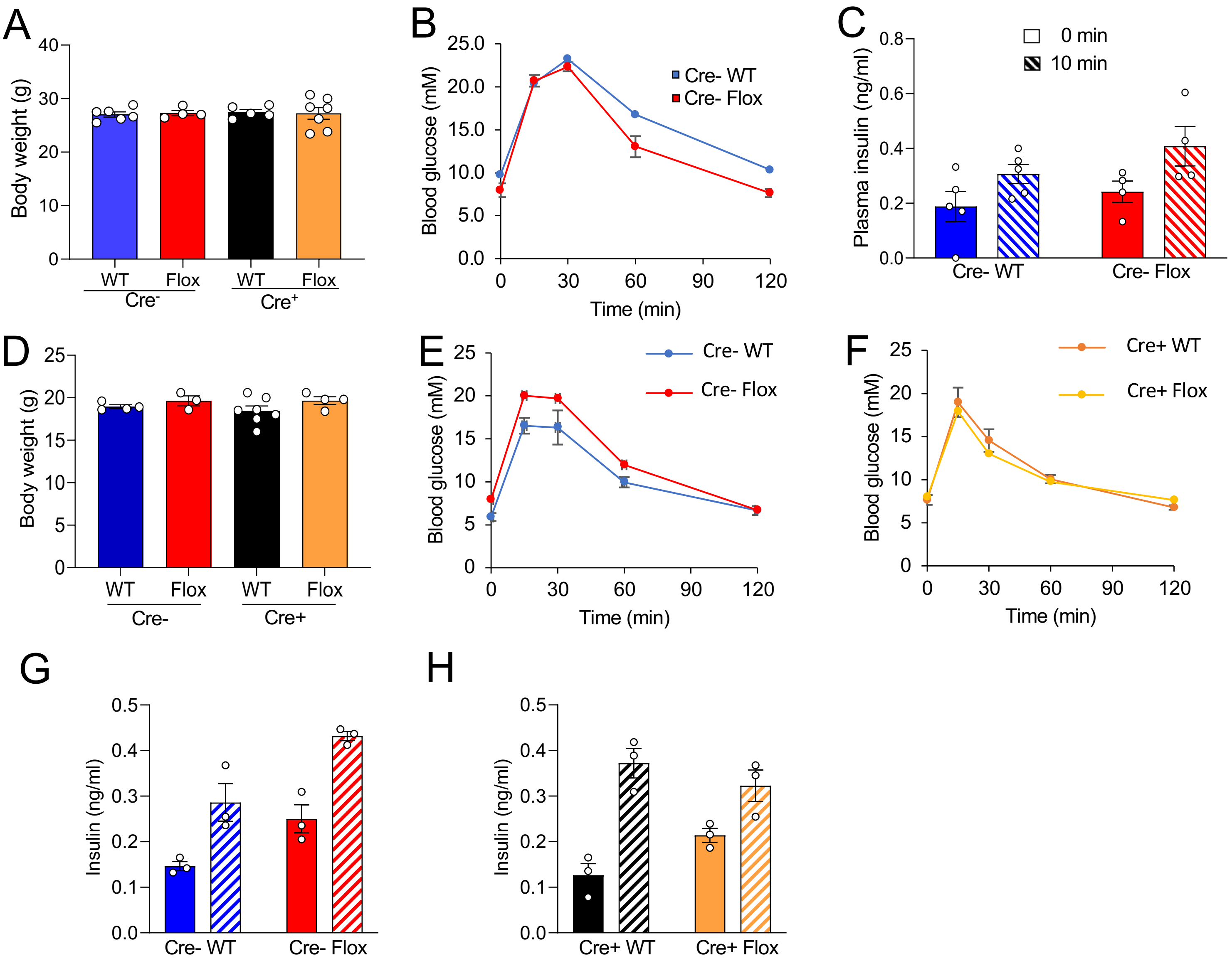

Supplemental Figure 2- β-cell Cre expression does not affect body weight, glucose tolerance, and glucose-stimulated insulin secretion in male and female mice.

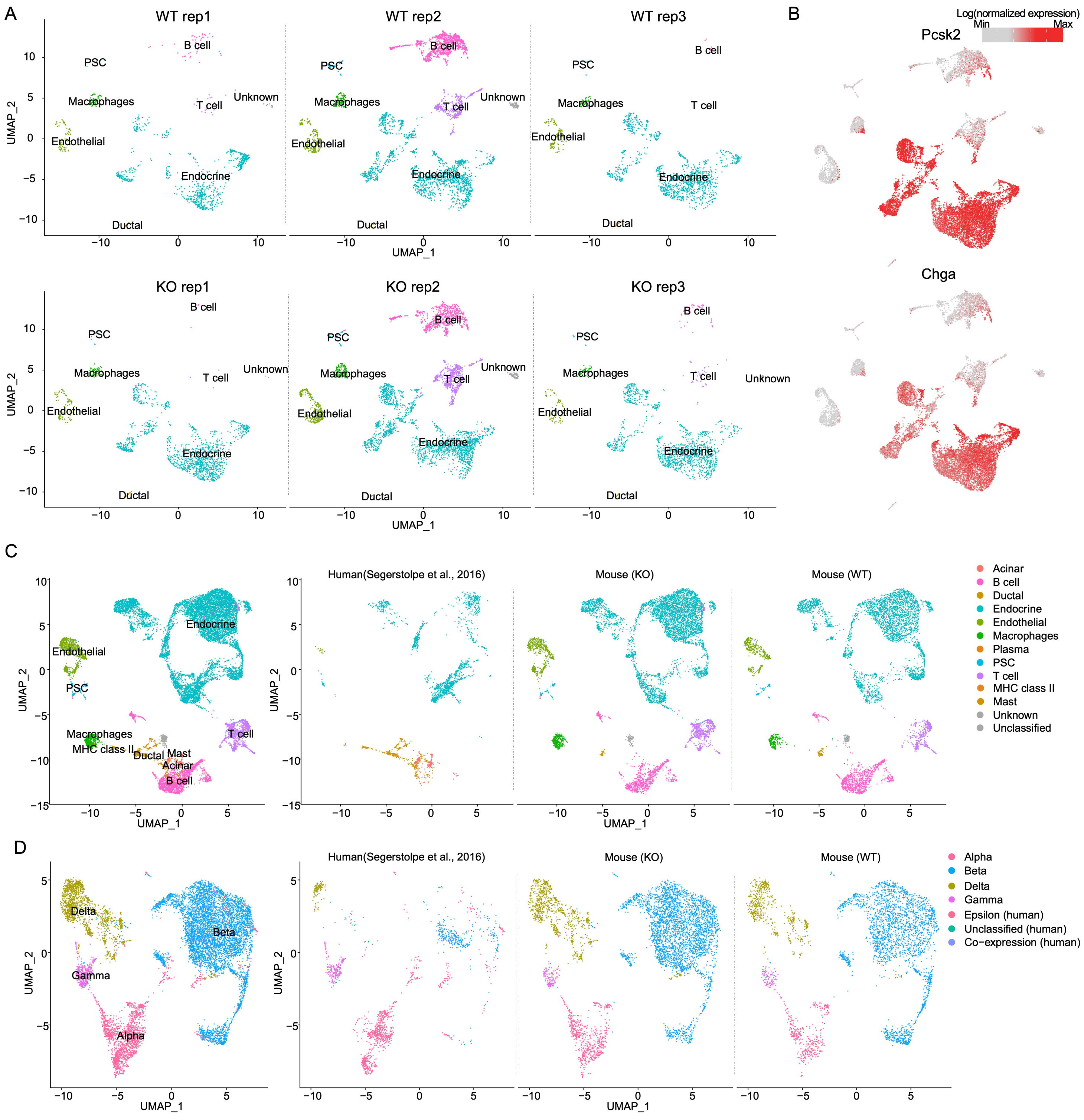

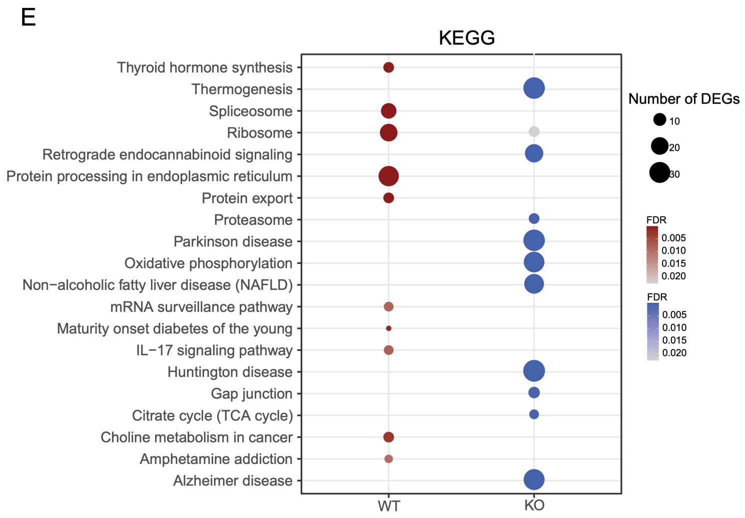

Supplemental Figure 3- Clustering of islet cells according to established markers.

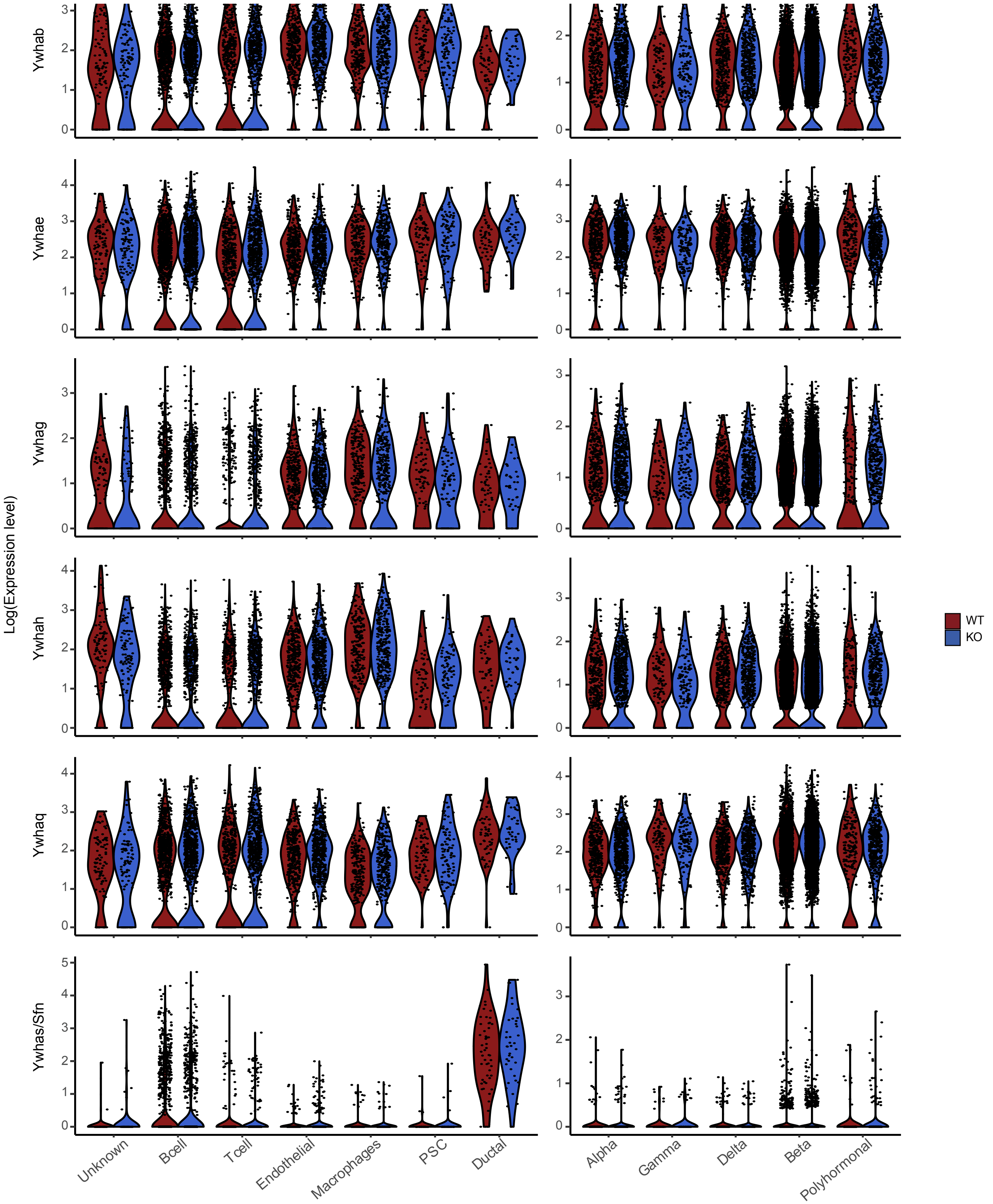

Supplemental Figure 4- No differences in *Ywhaz* mRNA levels were observed in non β-cells from islets of β14-3-3ζKO mice.

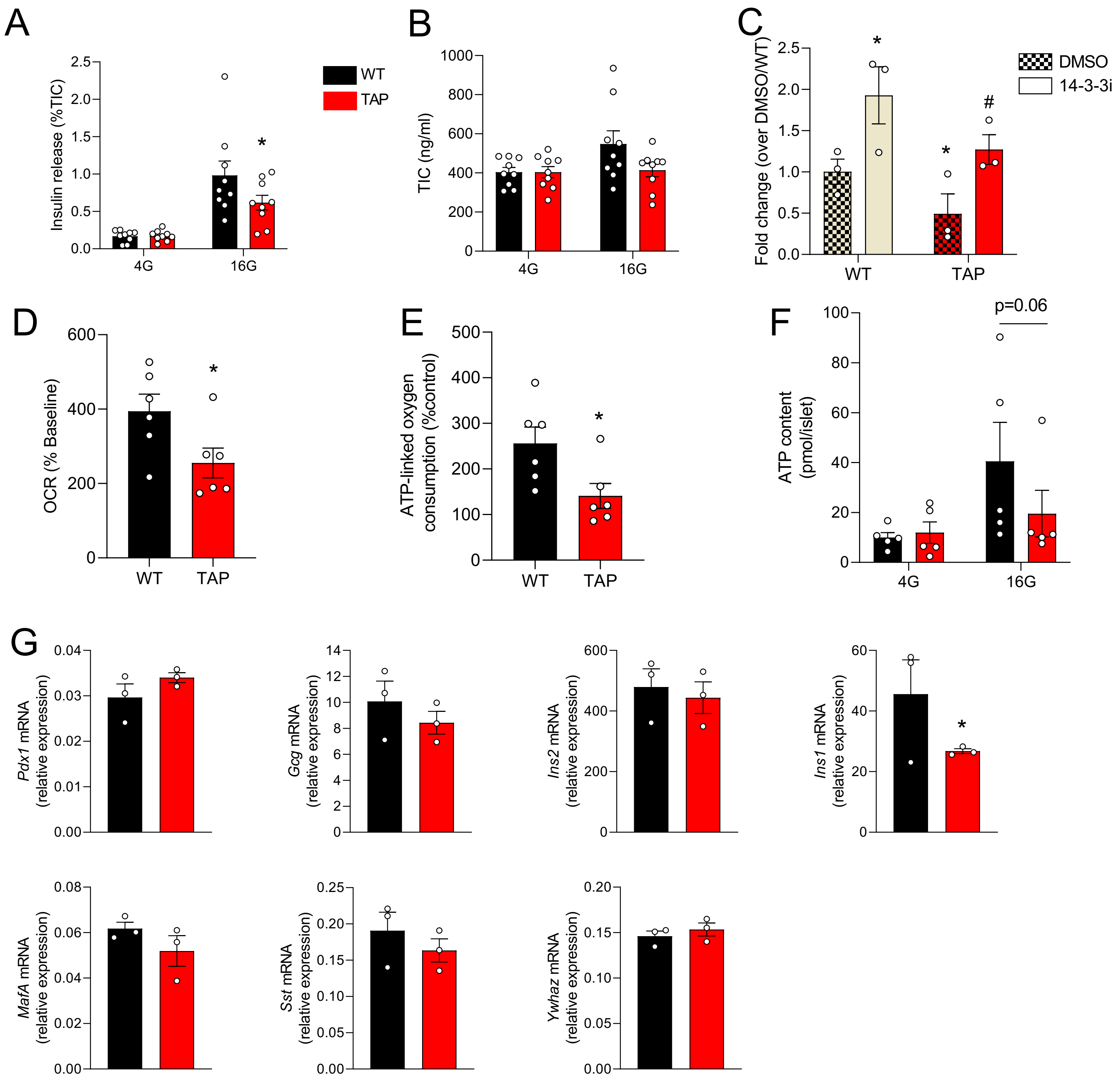

Figure S5. Over-expression of 14-3-3ζ in β-cells inhibits glucose-stimulated insulin secretion and mitochondrial function

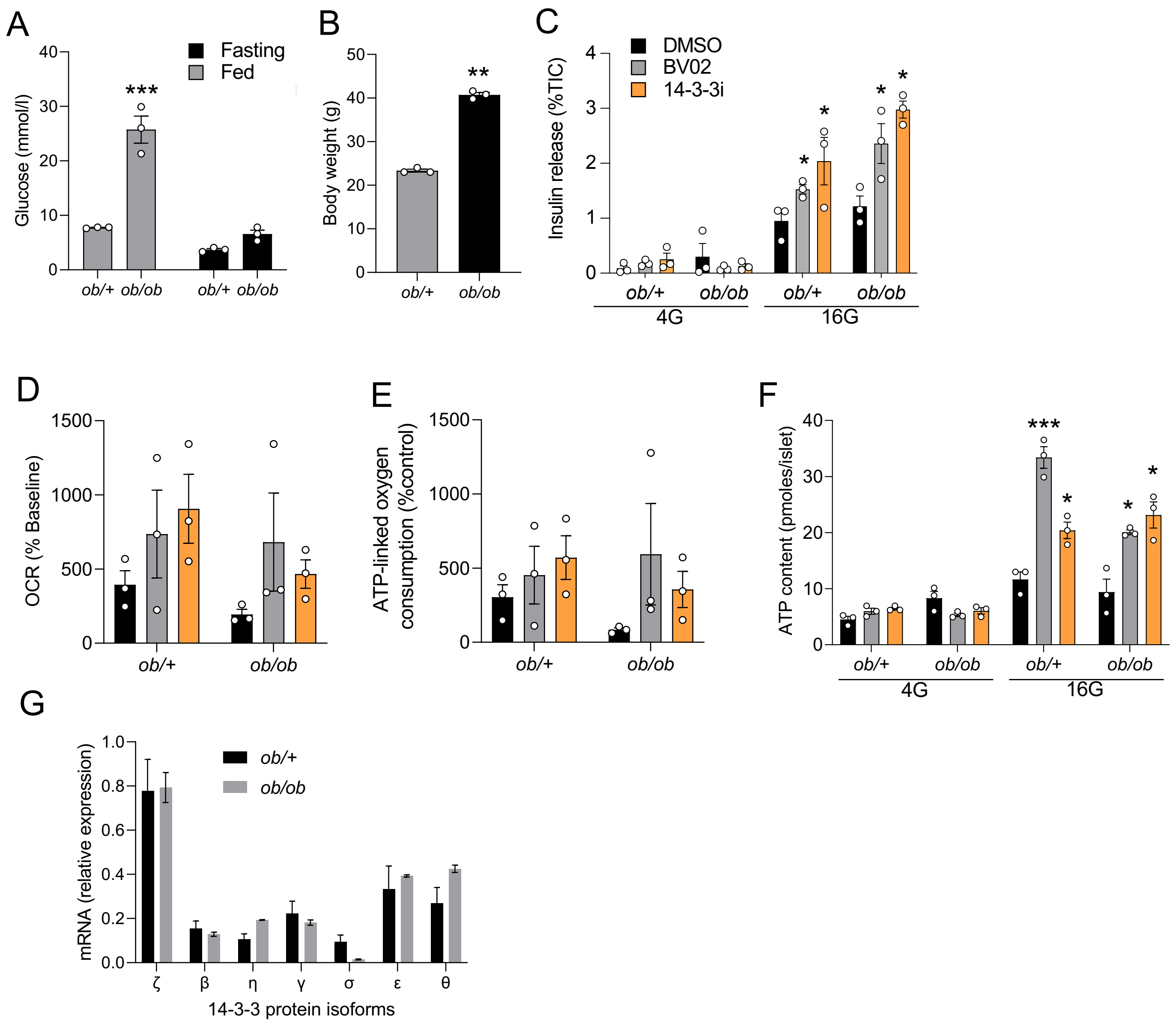

Figure S6. Effect of 14-3-3 protein inhibition on insulin secretion, mitochondrial function. and gene expression in islets of *ob/ob* mice.

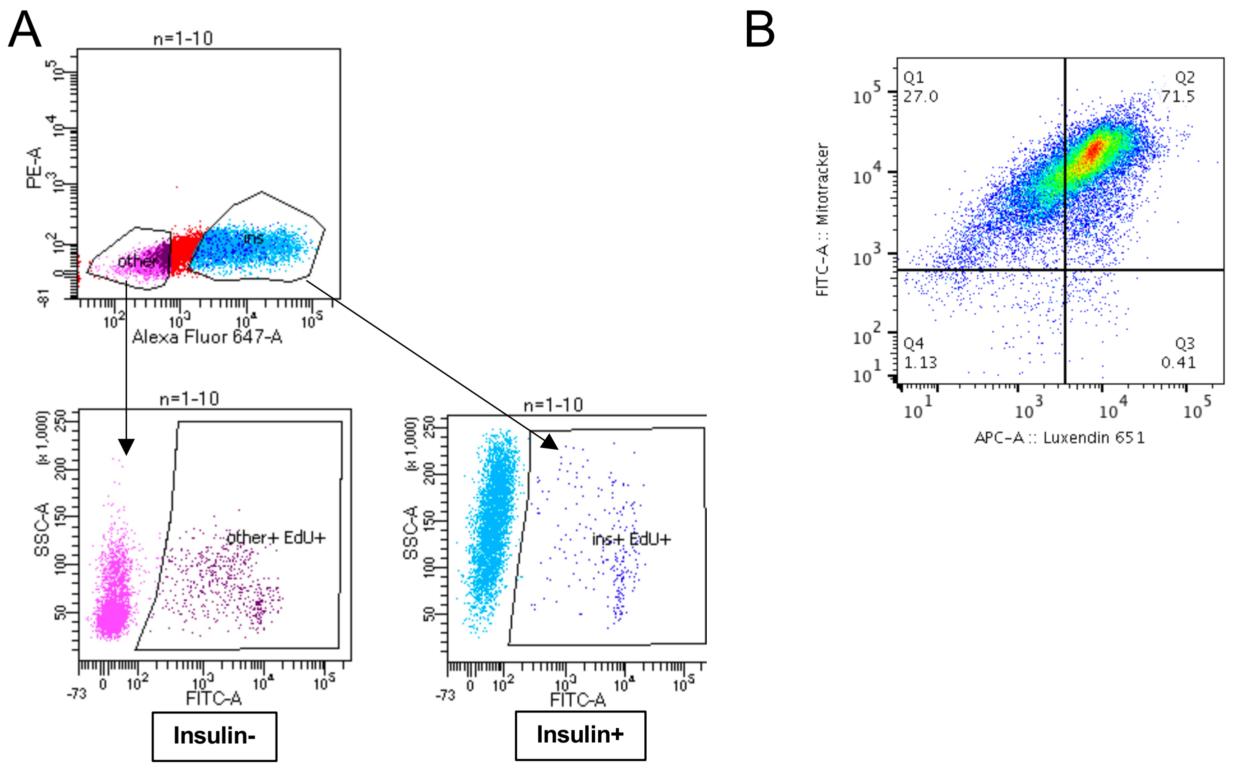

Supplemental Figure 7- Gating strategies used for Flow Cytometry.
